# Policymakers’ experience of a capacity-building intervention designed to increase their use of research: a realist process evaluation

**DOI:** 10.1186/s12961-017-0234-4

**Published:** 2017-11-23

**Authors:** Abby Haynes, Sue Brennan, Sally Redman, Anna Williamson, Steve R. Makkar, Gisselle Gallego, Phyllis Butow

**Affiliations:** 1 Sax Institute, 235 Jones Street, Ultimo, NSW 2007 Australia; 2 Sydney School of Public Health, Edward Ford Building (A27), University of Sydney, Camperdown, NSW 2006 Australia; 3 Australasian Cochrane Centre, School of Public Health and Preventive Medicine, Monash University, 553 St Kilda Road, Melbourne, Victoria 3004 Australia; 4 School of Medicine, University of Notre Dame, 160 Oxford St, Darlinghurst, NSW 2010 Australia; 5 Centre for Medical Psychology & Evidence-based Decision-making, University of Sydney, The Lifehouse, 119-143 Missenden Rd, Camperdown, NSW 2006 Australia

**Keywords:** Participant perspectives, Research utilisation, Process evaluation, Realist evaluation, Health policy

## Abstract

**Background:**

An intervention’s success depends on how participants interact with it in local settings. Process evaluation examines these interactions, indicating why an intervention was or was not effective, and how it (and similar interventions) can be improved for better contextual fit. This is particularly important for innovative trials like Supporting Policy In health with Research: an Intervention Trial (SPIRIT), where causal mechanisms are poorly understood. SPIRIT was testing a multi-component intervention designed to increase the capacity of health policymakers to use research.

**Methods:**

Our mixed-methods process evaluation sought to explain variation in observed process effects across the six agencies that participated in SPIRIT. Data collection included observations of intervention workshops (n = 59), purposively sampled interviews (n = 76) and participant feedback forms (n = 553). Using a realist approach, data was coded for context-mechanism-process effect configurations (retroductive analysis) by two authors.

**Results:**

Intervention workshops were very well received. There was greater variation of views regarding other aspects of SPIRIT such as data collection, communication and the intervention’s overall value. We identified nine inter-related mechanisms that were crucial for engaging participants in these policy settings: (1) Accepting the premise (agreeing with the study’s assumptions); (2) Self-determination (participative choice); (3) The Value Proposition (seeing potential gain); (4) ‘Getting good stuff’ (identifying useful ideas, resources or connections); (5) Self-efficacy (believing ‘we can do this!’); (6) Respect (feeling that SPIRIT understands and values one’s work); (7) Confidence (believing in the study’s integrity and validity); (8) Persuasive leadership (authentic and compelling advocacy from leaders); and (9) Strategic insider facilitation (local translation and mediation). These findings were used to develop tentative explanatory propositions and to revise the programme theory.

**Conclusion:**

This paper describes how SPIRIT functioned in six policy agencies, including why strategies that worked well in one site were less effective in others. Findings indicate a complex interaction between participants’ perception of the intervention, shifting contextual factors, and the form that the intervention took in each site. Our propositions provide transferable lessons about contextualised areas of strength and weakness that may be useful in the development and implementation of similar studies.

**Electronic supplementary material:**

The online version of this article (doi:10.1186/s12961-017-0234-4) contains supplementary material, which is available to authorized users.

## Background

This paper presents a realist analysis of how a novel, multi-component intervention trial designed to increase research use capacity, known as the Supporting Policy In health with Research: an Intervention Trial (SPIRIT), functioned in six health policy agencies. Data from a mixed-methods process evaluation is used to unpack the processes of engagement and participation that were hypothesised to mediate the intervention’s success. These intermediate impacts are conceptualised as process effects (see Box 1 for definitions). We do this by describing what was delivered in the intervention and what process effects were observed, then identify explanatory ‘Context + Mechanism → Process effect' configurations that show how the intervention, and the trial more broadly, was perceived by participants, why this varied across the participating organisations, and how these perceptions affected receptivity to the intervention’s ideas and resources. A realist approach is used because it supports rigorous comparative analysis of how those targeted by an intervention make sense of what it offers, and how this is shaped by context [1–3].

**Box 1:** Definitions of key concepts used in this paper

Context	In realist terms, context is any system, structure or condition that affects outcomes, including individuals’ attributes and social interactions [3]
Mechanism	Mechanisms are what makes an intervention work: “*They are not the observable machinery of program activities, but the response that interaction with a program activity or resource triggers (or does not trigger) in the reasoning and behaviour of participants*” [70]
Process effects	These are proximal impacts that influence intervention outcomes or are of evaluative interest for other reasons (e.g. they help explain unexpected variation in implementation); others use the term ‘formative outcomes’ [84]; Desired process effects are those the investigators consider to be prerequisites for a successful intervention
Programme theory	This is, “*An explicit theory or model of how an intervention contributes to a set of specific outcomes through a series of intermediate results*” [85]; programme theory should be plausible, useful and consistent with the evidence
Proposition	Propositions are generalised theoretical statements grounded in the data [86]; in realist evaluation, they link and condense information about contexts, mechanisms and outcomes; propositions are refined through empirical testing but remain fallible [87]
Realist process evaluation	Process evaluation helps explain how an intervention had its effects [7]; realist process evaluation applies realist principles to this process and investigates causal patterns (known as demi-regularities) to show how intervention strategies may be operating under what conditions to generate process effects for which groups [3]
Retroduction	This is a form of analysis that “*involves constant shuttling between theory and empirical data, using both inductive and deductive reasoning*” [88]

### Understanding interventions

Interventions – planned activities to change individual, group and/or organisational behaviour – are not passively received, but are actively shaped by the people who participate in them and the circumstances in which they are delivered [4–6]. Understanding the ways in which participants interact with and perceive an intervention is vital for determining how and why it was, or was not, effective [7]. This requires moving beyond measures of participant satisfaction – sometimes derided as “*happy face evaluation*” [8] – towards methods which delve into “*the complexity, flux and contextual variation that inevitably occurs in real life situations*” [9].

Many organisational capacity-building interventions fail because they do not take sufficient account of participants’ workplaces [10]. Successful interventions introduce strategies (ideas, activities and resources) that are contextually apt [7, 11] and which are therefore able to produce desired interactions [3]. For example, in organisational interventions, participants’ perceptions and interactions are affected by factors such as the organisation’s culture [12], its history of change [13, 14], staff heterogeneity [15] and trust in management [13].

Information about how implementation interacts with people and place over the course of an intervention is frequently overlooked [16]; yet, it is necessary for making informed assessments about the worth, adaptability and transferability of strategies designed to bring about individual or organisational change [9]. In multi-component interventions it is often impossible to disentangle which components were more or less effective, or what variations in combination might maximise effectiveness [17]. These interventions frequently trigger unanticipated causal processes and have unpredictable impacts that standardised measures are unlikely to capture [18]. This may be especially important for interventions where participants have involvement in the tailoring and/or delivery of an intervention, since their attitudes towards its content, form and goals are likely to have profound impacts on what is delivered and how it is received [19, 20]. Indeed, there is an established link between outcomes and the ways that participants gauge the quality of their involvement in tailoring the scope, content and process of flexible interventions [4].

Context-sensitive design, implementation and evaluation are particularly pressing for interventions that attempt to increase the use of research in policy processes. Policymaking is “*a contested arena of negotiation…. messy, complex, and serendipitous*” [21], (where research, and researchers [22]), are used strategically [23, 24]. Macro-level political and institutional factors influence how policymakers and policy organisations engage with and make use of research [23], and will therefore mediate their relationships with research utilisation interventions. Given that the use of research is cultural and rhetorical as well as technical [25], where an intervention promotes greater use of research, or claims to be evidence based, participants may actively critique that premise [26, 27]. Thus, determining if and how such an intervention is compatible with participants’ beliefs and practice norms is critical.

Despite these arguments, many interventions are reported (and, by implication, conducted) with minimal consideration of the interactions between the intervention activities, the people who took part, and the circumstances that mediated this relationship [9, 28]. As Clark et al. note, “*Little research has explored individuals’ experiences of programmes or examined how programme dimensions lead to changes in behaviour. …individuals’ meanings, experiences and reactions to the programme and the effects of their wider context are simultaneously disregarded*” [29]. Realist process evaluation is well equipped to redress these oversights [1, 3].

### The study being evaluated: SPIRIT

SPIRIT was a stepped wedge cluster randomised trial that tested the effects of a novel intervention designed to increase the capacity of health policy agencies to use research. Six organisations in Sydney, Australia, participated. Five were state government agencies and one was a national organisation funded by the federal government. An agency was eligible to participate if (1) a significant proportion of its work was in health policy or programme development, and (2) there were at least 20 staff involved in health policy, programme development or evaluation. A sampling frame was drawn from Government websites that listed all New South Wales and Australian government health policy and programme agencies located in Sydney. Members of the investigator team reduced this list to 16 potentially eligible agencies and ranked as highest those with the greatest focus on health and the largest numbers of relevant staff. The top six agencies were invited to take part, and all agreed [30]. Each agency’s Chief Executive Officer (CEO) signed an organisational-level agreement to participate in SPIRIT and nominated a liaison person: an internal member of staff who would be responsible for coordinating SPIRIT in their setting for the duration of the trial. There were six rounds of outcome data collection using three evaluation tools. These are described in detail elsewhere [30–35].

**Fig. 1:**
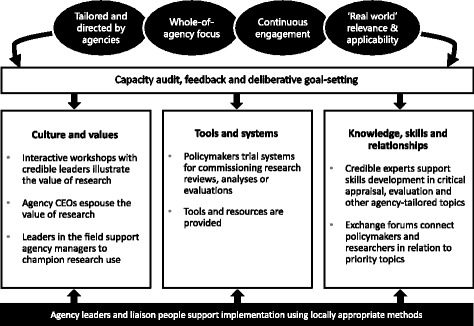
SPIRIT intervention model

The intervention aimed to increase agency capacity to use research in relation to three goals, namely (1) the organisation and staff value research more; (2) more tools and systems are in place to support research engagement actions and the use of research; and (3) staff have greater knowledge and skill in research engagement actions and the use of research. SPIRIT’s design was informed by an action framework [36] and underpinning change principles that reflected composite theory from psychology, organisational science, adult learning and the research utilisation literature [30]. The intervention comprised multiple components hinging on interactive workshops such as research skills seminars, exchange forums with researchers, and a leadership programme targeting senior managers. Other activities included the provision of tools and resources (such as an online research portal); practice using systems for commissioning research reviews, analyses or evaluations; and CEO espousal of research-informed policymaking (Fig. 1). Agencies could choose options within and tailor many of the components to address local priorities. Each agency was asked to identify two lists of potential participants, namely (1) all staff involved in policy or programme development, implementation or evaluation who would be invited to take part in intervention activities and data collection and (2) managers who would take part in the leadership programme and promote SPIRIT.

An onsite introductory information session preceded the intervention and data collection in each site. The round of data collection that took place immediately before the intervention functioned as an audit and was followed by a feedback forum in which the lead investigator facilitated a deliberative dialogue with leaders about their agency’s findings. Intervention goals targeting research engagement and use were identified during this process. Agency leaders considered how they would like to use SPIRIT’s options to address these goals and, if applicable, any additional (non-SPIRIT) strategies for reaching their goals.

External research and policy experts were contracted to deliver workshops. They were briefed on SPIRIT’s ‘change principles’ and their workshop’s objectives. The content of the tailored workshops was negotiated with the agency’s liaison person, with input from presenters. Members of the SPIRIT research team coordinated the development and delivery of workshops and other intervention activities. Each site had a dedicated knowledge broker from the SPIRIT team who acted as the onsite ‘face’ of SPIRIT, negotiated tailoring and attended all intervention activities.

An in-depth, mixed methods process evaluation informed by realist thinking was conducted in parallel with the intervention. This paper is based on that data.

### The role of process evaluation

Process evaluation investigates an intervention’s implementation, change mechanisms and contextual interactions in order to explain (insofar as this is possible) how and why the intervention functioned as it did in each intervention site [18]. Process evaluation does not determine whether study outcomes are achieved, but it can identify process effects, namely proximal impacts of an intervention that make achieving outcomes more or less likely [37].

### Aims

Using a realist evaluation approach [1, 3, 38, 39], we aimed to generate transferable learning in relation to the questions, (1) To what extent did SPIRIT achieve the desired process effects in each agency? and (2) How were these process effects generated? i.e. What mechanisms seem to account best for the patterns of engagement and participation observed across all agencies?

## Methods

### Realist evaluation

The SPIRIT process evaluation comprised a fidelity assessment and a theory-driven exploration of the interaction between the intervention, participants and the implementation circumstances, with the expectation that this would probably take a different form in each of the six agencies [40]. Theory-driven evaluation seeks to uncover causal pathways [41] and is well suited for understanding how multicomponent interventions function in complex real-world settings [42]. In this study, we adopt a particular theory-driven approach – a realist evaluation [43] – following the methods associated with Pawson [1], Pawson and Tilley [3], and others in the RAMESES II project [39]. Realist evaluation focuses on an intervention’s underlying theory as its unit of analysis [1, 3], with the aim of determining “*what works, for whom, in what circumstances, and how*” [3, 44]. Realists posit that interventions introduce ideas and opportunities that generate effects in conjunction with participants’ reasoning and resources. Thus, the interaction between intervention activities and the contexts of each intervention site will determine what (if any) mechanisms are activated and what outcomes (intended and unintended) are generated [45, 46].

We used a realist approach because it maximises the transferability of findings and operational learning from one setting to another (an enduring concern in intervention evaluation [47]), while also recognising complexity and the need to look beyond one-size-fits-all ways of responding to problems [1, 3, 48, 49]. Realist evaluation has been used effectively in studies of policy processes [50], implementation research [51], knowledge exchange [52] and evaluations of flexible intervention trials [19, 29], making it especially suitable for addressing the methodological challenges presented by a multi-component, novel and theoretically eclectic trial like SPIRIT (outlined in detail elsewhere [53]).

Importantly, analyses arising from realist evaluations are tentative, claiming only to be an informed hypothesis of “*how something might be*” [54] rather than a definitive version of reality. These hypotheses accrue plausibility when tested in further studies, but remain open to revision or rejection if alternative theories are more convincing [45]. In our study, data collection, management and analysis were concurrent; thus, we were continually testing and revising hypotheses within and across the six intervention sites over the 30-month study, but our findings are embryonic in realist terms.

### Initial programme theory

Realist evaluation develops, tests and refines programme theory. SPIRIT was informed by a mixture of formal theory and experiential knowledge [30], and had both a well-articulated action framework [36] and clear principles about what should be provided [53], but did not offer hypotheses about the mechanisms that would generate increased capacity to use research. Based on existing trial materials and discussions with the designers, we articulated the overarching programme theory to make the intended causal pathway more explicit so that we could critique the assumptions underpinning the intervention design [1, 3, 55]. This was refined and agreed through further consultation:

*SPIRIT will engage and motivate agency leaders to ‘own’ the intervention using audit feedback, deliberative goal-setting and program tailoring. This agency-driven approach will generate a priority-focused program that offers locally relevant practice support and accommodates differences in agencies’ values, goals, resources and remits. The program will comprise a suite of andragogical activities, tools, and connection across the research-policy divide that provide resources and build knowledge, skills and relationships. It will be supported via modelling and opinion leadership by agency leaders and dynamic external experts. CEOs will promote SPIRIT in their agencies and liaison people will facilitate the tailoring and implementation. These strategies will act synergistically to stimulate and resource participants at different organisational levels, leading to changes in values, practice behaviours and agency processes. This will facilitate increased use of research in policy processes.*

This pathway informed the data collection, providing pointers about what to look for, but was used flexibly (rather than as a rigid investigative framework) as befits an exploratory study. We also looked for unintended effects, and considered alternative causal pathways that might better explain observed effects. The data offered the opportunity to develop a much richer understanding of the social processes and interactions than had previously been possible.

### Process effects

The programme theory was used to identify desired process effects via discussion with the study designers. We then explored how these process effects were achieved in each setting for the range of targeted participants, or why they were not. Our conceptual framework for this work was informed by the implementation science literature that focuses on social processes and interaction in interventions (e.g. [6, 26, 56–60]).

### Data collection

Causation, and the mechanisms that generate it, are seldom observable [3]. Therefore, in realist evaluation, data is triangulated to identify the interactive patterns that can most plausibly explain how the intervention led to the observed outcomes [61]. Quantitative data is helpful for identifying outcomes [1], while qualitative methods are usually necessary “*to discover actors’ reasoning and circumstances in specific contexts*” [62]. We used the following methods to capture information:

- Semi-structured interviews with 5–9 participants from each agency early in the intervention period (n = 33) and post-intervention (n = 43). Interviewees were purposively selected for maximum variation in work roles, attitudes to research and experiences of SPIRIT in order to explore the breadth of dimensions expected to influence interactions with the intervention [7]. Open-ended questions and prompts explored interviewees’ work practices and contexts, and their experiences and perceptions of SPIRIT, including their explanations for any change. The interview questions are available elsewhere [40]. This combination of context-, causal- and impact-focused questions across diverse participants was used to refine theory about what was working (or not), for whom and in what circumstances.
- Observations of intervention workshops (n = 59), and informal opportunistic conversations with participants before and after workshops. Workshops were audio recorded and field notes were written immediately afterwards. A checklist was used for fidelity coding through which we monitored the extent to which ‘essential elements’ of the intervention were delivered (detailed elsewhere [59]).
- Anonymous participant feedback forms (n = 553). These comprised Yes/No ratings on six statements: (1) The workshop was interesting, (2) The workshop was relevant to my work, (3) The workshop was realistic about the challenges and constraints of our work, (4) The presenter had appropriate knowledge and skills, (5) It is likely that I will use information from this workshop in my work, (6) It is likely that SPIRIT will benefit my agency (Additional file 1). Some workshops had additional items, e.g. the forms for audit feedback forums included items about the clarity of the data and participants’ confidence that SPIRIT would be adequately tailored for their agency. All forms contained three open-ended questions: (1) ‘What worked well?’, (2) ‘What could be improved?’ and (3) ‘Any other comments?’ Forms were distributed prior to intervention workshops and completed immediately afterwards.
- Formal and informal interviews with the people implementing SPIRIT and the commissioned presenters*.*
- Limited access to information from the interviews conducted as part of SPIRIT’s outcome evaluation. These interviews focused on (1) organisational support for research use (n = 6), and (2) the role of research in the development of a recent policy or programme (n = 24). We reviewed transcripts from the first round of interviews (prior to the intervention), but thereafter were blinded to this data so that it would not influence the ongoing process evaluation analysis.

### Data management and analysis

#### Qualitative data

Data was initially analysed for the whole process evaluation. Interview data was managed using framework analysis [63] within NVivo v.10 [64] and used to develop descriptive case studies [65] in combination with data from the fidelity assessment, running memos for each agency, interviewee memos, the thematically coded data from field notes and the open-ended questions in feedback forms. These case studies described (1) each agency’s context, change trajectory, workforce and practice norms, (2) their research use practices and culture, (3) how SPIRIT was implemented in each setting, and (4) the interactions between (1), (2) and (3). Framework categories and the structure of the case studies were iteratively developed from *a priori* concerns (such as the constructs the intervention was targeting and the hypothesised causal pathway), and from themes identified using inductive analysis [66, 67]. The method of constant comparison [68] was used to query and refine the initial programme theory and other emergent hypotheses throughout the trial. This work is described in more detail elsewhere [40].

#### Quantitative data

For each agency, we calculated the number and percentage of feedback forms responding ‘Yes’ to each of the six statements outlined earlier. In calculating these frequencies, the four different types of workshops (symposia, research exchanges, leaders’ forums and audit feedback forums) were aggregated.

#### Realist analysis

Using the data described above, we sought to explore the hypothesised pathway identified in the initial programme theory and to identify any other pathways leading to the interventions’ observed process effects, plus other impacts reported by participants or members of the implementation team [42].

We employed a retroductive analytical approach that attempts to explain phenomena by theorising about what mechanisms are capable of producing them [69]. This involves studying events “*with respect to what may have, must have, or could have caused them. In short it means asking why events have happened in the way they did*” [51]. In accordance with realist evaluation principles, we focused on the interaction of SPIRIT with features of each agency’s context that appeared most likely to have influenced process effects [42, 70]. We developed explanatory configurations of the patterns we saw in the data. In realist evaluation, these are typically called Context + Mechanism → Outcome configurations [1, 3], but because the ‘outcomes’ of interest in process evaluation are process effects rather than study outcomes, we have called them Context + Mechanism → Process effect configurations herein. Propositions were then developed to summarise each configuration. This work depended on using each type of data to query, explain and balance the other to reach as comprehensive as possible accounts of what happened and why [71, 72]. Original data sources were revisited as required.

These process effects were identified prior to the development of Context + Mechanism → Process effect configurations and were used as a starting point in much of the analysis – although realist evaluation depicts outcomes (or, in our case, process effects) as the final step in the sequence, the analysis tends to start by identifying effects, then working backwards to investigate the conditions (context and mechanisms) that caused them [73]. We traced connections to and from observed process effects asking ‘What caused this?’, ‘Why didn’t this unfold as anticipated?’ and ‘What best explains these different responses between agencies?’ Analysis involved looking for data that might indicate the absence or weak functioning of mechanisms as well as the presence of a mechanism. This was aided by Dalkin et al.’s [46] assertion that mechanisms may vary in intensity rather than simply being present or absent.

AH, who led the process evaluation, reviewed and coded all data sources. SB, who contributed to the process evaluation design and analysis throughout the trial, independently reviewed a proportion of interview transcripts and cross-agency fieldwork memos. Their preliminary Context + Mechanism → Process effect configurations overlapped extensively and were workshopped with further reference to the wider data set to develop agreed configurations. Further discussion with our co-authors resolved differences and refined the final findings.

This analysis relied on abductive reasoning [74], which is an iterative cycling between data and likely explanations that incorporates inductive and deductive processes. We looked for evidence of factual causal mechanisms, and for evidence that supported, discounted or nuanced current causal hypotheses both in real time (as the intervention unfolded) and retrospectively (reviewing data already collected). Throughout this process, we sought to identify where our evolving Context + Mechanism → Process effect configurations aligned with existing theory; we revisited the theories used to inform the development of SPIRIT, asking to what extent did these theories support the patterns we were observing in the data, and also considered other theories that might better explain our findings. See Additional file 2 for an overview.

## Results

In this section, we describe the implementation of the SPIRIT intervention, outline the observed process effects, and then attempt to explain how these effects were generated using Context + Mechanism → Process effect configurations. Finally, we present the revised programme theory.

### Implementation

As Additional file 3 shows, some aspects of SPIRIT were delivered with a high degree of implementation fidelity; indeed, every agency received audit feedback and the intended number of components on the topics they requested. Intra-organisational processes that were outside the control of the implementation team had greater variation. The promotion of SPIRIT and much of its administration depended on the attitudes and behaviours of liaison people and each organisation’s leaders, and to a lesser extent, the expert presenters commissioned for each workshop. This resulted in some loss of SPIRIT’s theoretical fidelity, i.e. the extent to which the intervention delivered its ‘essential elements’ (these are discussed in more detail elsewhere [53]). For example, the essential elements stipulated that workshops should be non-didactic and therefore the presenters should encourage participants to contribute as much as possible. Many workshops were highly interactive, such as the deliberative audit feedback forums, but others were not. This was because (1) the expert presenters sometimes overrode their briefing to facilitate discussion; (2) liaison people occasionally tried to maximise value by cramming content into workshops, which limited opportunities for participation; and (3) unexpectedly, the agencies seldom took up offers to co-design and co-present workshops.

In some sites, SPIRIT’s reach was constrained more than anticipated. Agency 6, for example, chose to focus some components of the intervention on one group of staff and limited participation accordingly. In Agency 3, managers attempted to minimise the onerousness of data collection by excluding some eligible staff from invitations to complete surveys. Agencies also defined their leadership groups quite differently, resulting in wide variation in the numbers and organisational roles of participants in the leaders’ programme.

### Process effects

Table 1 describes SPIRIT’s process effects, i.e. the actions, behaviours and responses hypothesised to be necessary for SPIRIT to generate the capacity-related outcomes measured in the trial. Column 1 lists the process effects both for the intervention and the trial evaluation; we include the latter because of their impact on the quality of the evaluation and the way that SPIRIT as a whole was perceived. Column 2 presents a summary of our observations about the extent to which these process effects occurred. Column 3 shows the data sources for our observations.

**Table 1:** Overview of SPIRIT’s process effects and data sources

Desired process effects for the trial	Observed process effects	Supporting data sources
1. Leaders espouse SPIRIT and its goals	All CEOs disseminated initial information about their agency’s participation in SPIRIT, but only four had a continuing visible role in supporting the intervention, e.g. sending updates and attending workshops; some executive members participated in each site, but to very different extents ranging from a half hour ‘drop in’ to repeated and enthusiastic participation; many managers talked about SPIRIT in team meetings and encouraged their staff to attend	Interviews at two time points (early-intervention ‘context’ and post-intervention ‘perceptions and impact’), ad hoc conversations with participants
2. Liaison people facilitate the intervention effectively	The use of a liaison person was very effective in the sites where the liaison person was enthusiastic about SPIRIT; four of the six worked hard to promote, tailor and administer the intervention, harnessing insider knowledge and using creative strategies, whereas the other two did not tailor or promote the intervention as thoroughly and expressed negative views to colleagues about SPIRIT	Observations of workshops, interviews and conversations as above, feedback from the SPIRIT team about their communications with liaison people
3. Targeted policymakers participate in, and are receptive to, intervention activities	Participation levels were good in that they met the SPIRIT team’s expectations for each site; each agency targeted different groups for different components so proportions and types of participants varied, but liaison people were satisfied with attendance and were occasionally surprised by very high numbers; attendance at workshops averaged between 11 and 20 participants per workshop, with between 102 and 158 total occasions of attendance across the six sites; there was full participation in other activities (e.g. trialling the commissioned research services); receptivity varied tremendously within, but especially between, agencies: see next section for more details, including possible reasons	Quantitative fidelity data from observations (using check lists and sign-in sheets), observations, interviews and conversations as above
4. Participants actively contribute to the content of those activities	Where there was opportunity, participants contributed greatly to workshop content via questions, discussion and case examples; interactivity was limited on some occasions in all agencies, usually because the presenter provided few opportunities; in larger groups, more senior staff tended to dominate, but other participants said this was still useful. Some liaison people helped craft workshop content and provided agency-based case examples; one agency co-presented a workshop; the agency staff nominated to test the research commissioning service were actively involved	Observations of workshops, including descriptive accounts of interactions and dynamics
5. Participants identify potentially useful ideas, techniques and/or resources	94% of those who completed a feedback form said they found workshops to be both relevant to their work and realistic about policy challenges and constraints; many interviewees identified specific benefits from SPIRIT, including improved awareness of useful researchers and research resources, understanding of the evidence relating to a policy problem and access to existing agency resources	Participant feedback forms, observations of workshops, interviews and ad hoc conversations with participants and liaison people
6. Participants use, or plan to use, these ideas, techniques and/or resources	Workshops facilitated less discussion than intended about how learning might be applied, but 95% of participants who completed a feedback form agreed, “*It is likely that I will use information from this workshop in my work*”; some interviewees said they planned to use ideas or resources, and a few had done so, especially newer staff; three liaison people had managerial-approved plans underway for research-focused education and/or systems improvement, e.g. mandated consideration of research in policy proposals; two agencies had plans to use their commissioned research products	
Desired process effects for the evaluation	Observed process effects	Supporting data sources
7. Liaison people facilitate data collection effectively	All liaison people facilitated data collection sufficiently, although it was occasionally delayed and required prompting; where liaison people championed SPIRIT they used additional strategies to encourage participation in data collection, in one agency this achieved a 100% response rate	Outcome measures completion figures, interviews with participants and liaison people, feedback from SPIRIT team
8. Targeted participants take part in data collection	In all agencies, there was full participation in the two interview-based measures, but more variable responses to the anonymous online survey; response rates dipped in the second measurement point, but stabilised after the survey was shortened; overall, the online survey response rate was 56% and there was a mean 74% response rate for process evaluation feedback forms; only three-quarters of invitees took part in a process evaluation interview	Outcome measures completion figures, interviews with participants and liaison people
9. The benefits of the intervention are judged to outweigh the burdens of the trial	Interviewees differed considerably in their assessments of the intervention, but where they felt it had benefits these were deemed to outweigh the trial’s burdens, this included those liaison people who championed SPIRIT from the start; workshops with high profile and dynamic ‘service-orientated’ presenters were especially valued; nearly 98% of all feedback form respondents agreed with the statement, “*It is likely that SPIRIT will benefit my agency*”	Early-intervention and post-intervention interviews, ad hoc conversations with participants and liaison people, feedback form data

### How were these process effects generated?

**Fig. 2:**
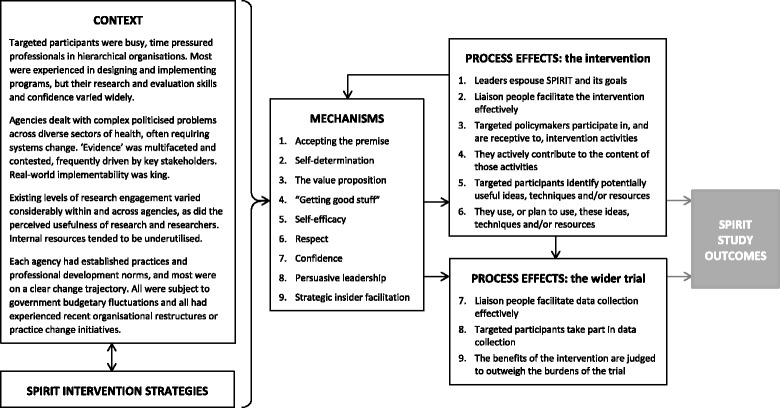
Overview of context-mechanism-process effects in the SPIRIT trial

We identified nine primary causal mechanisms (Fig. 2). The Context + Mechanism → Process effect configurations for each mechanism are presented in the following section. Each of the configurations begins with an overview of the context pertaining to that mechanism, a description of how we believe the mechanism functioned, how it generated process effects and how process effects differed between participating agencies. A proposition that summarises the hypothesised casual pathway precedes each configuration.

Cross-references to other mechanisms are in shorthand so that mechanism 1 reads as M1, etc. Similarly, agency numbers are shortened so that Agency 1 is shown as A1, and so on. Inevitably, this is a highly truncated presentation of our findings. For those who seek more detail, a narrative description of the data that informed our identification of each mechanism can be found in Additional file 2. This additional file provides an ‘evidence link’ between the data and the findings that follow.

#### Mechanism 1

Accepting the premise (Table 2)

**Table 2:**
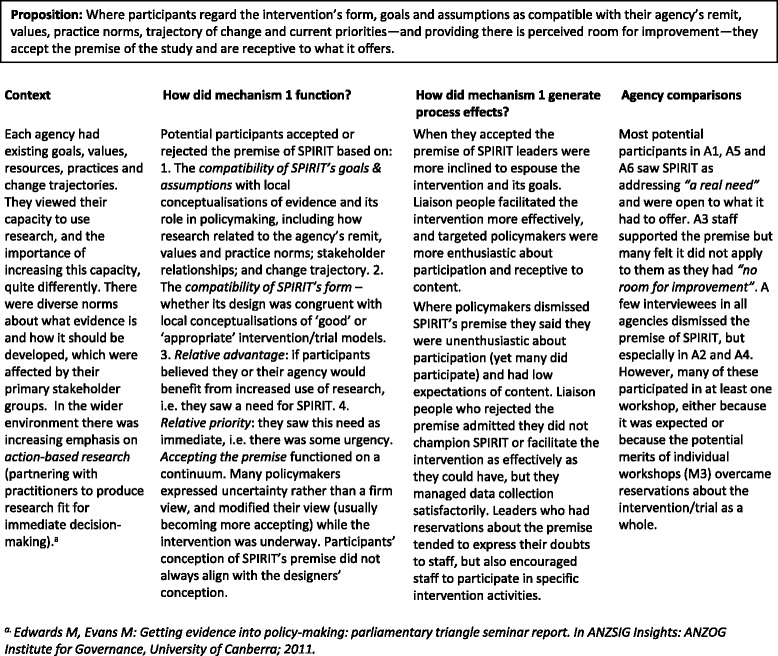
Mechanism 1 - Accepting the premise

#### Mechanism 2

Self-determination (Table 3)

**Table 3:**
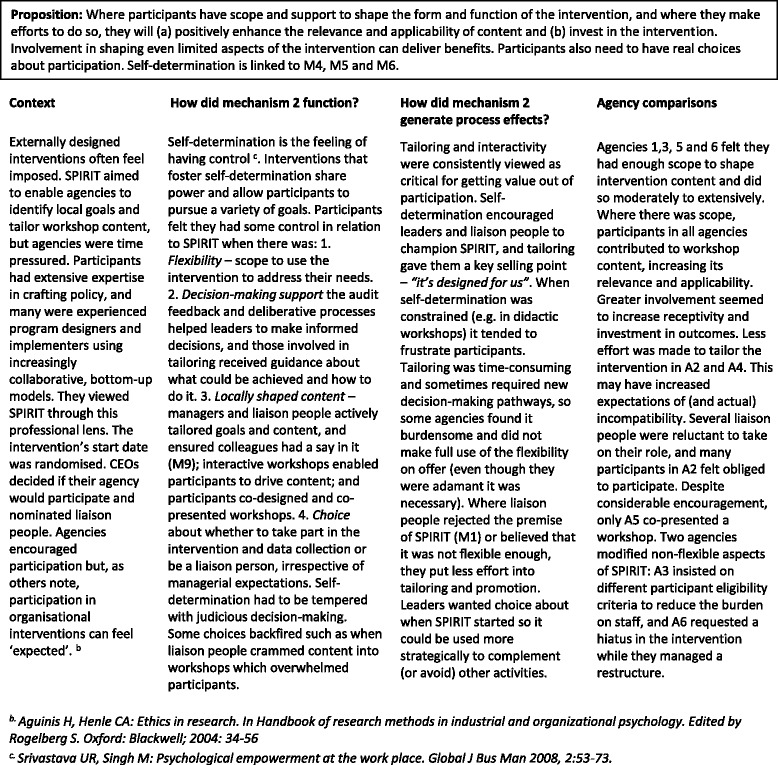
Mechanism 2 – Self-determination

#### Mechanism 3

The value proposition (Table 4)

**Table 4:**
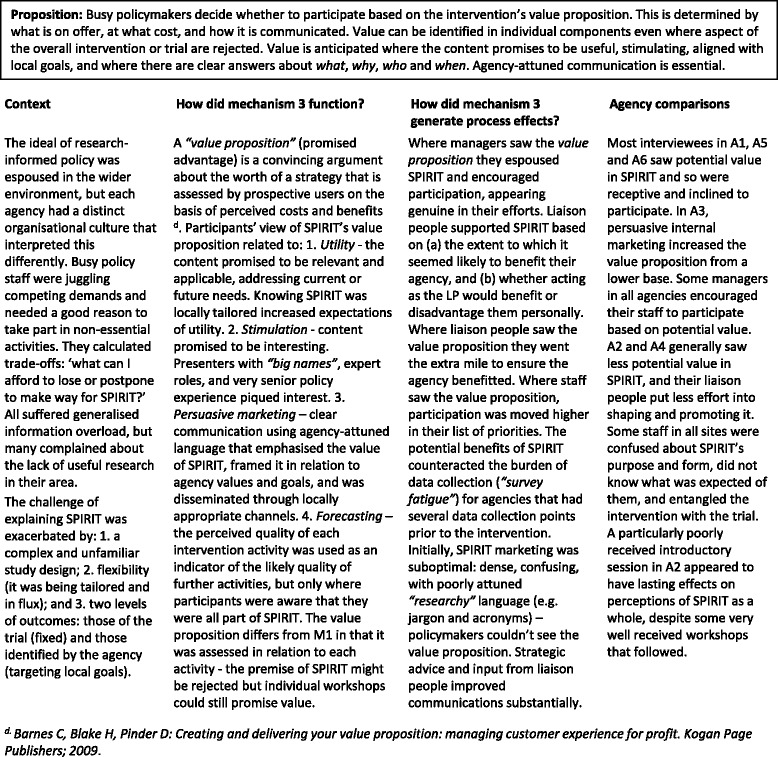
Mechanism 3 – The value proposition

#### Mechanism 4

“Getting good stuff” (Table 5)

**Table 5:**
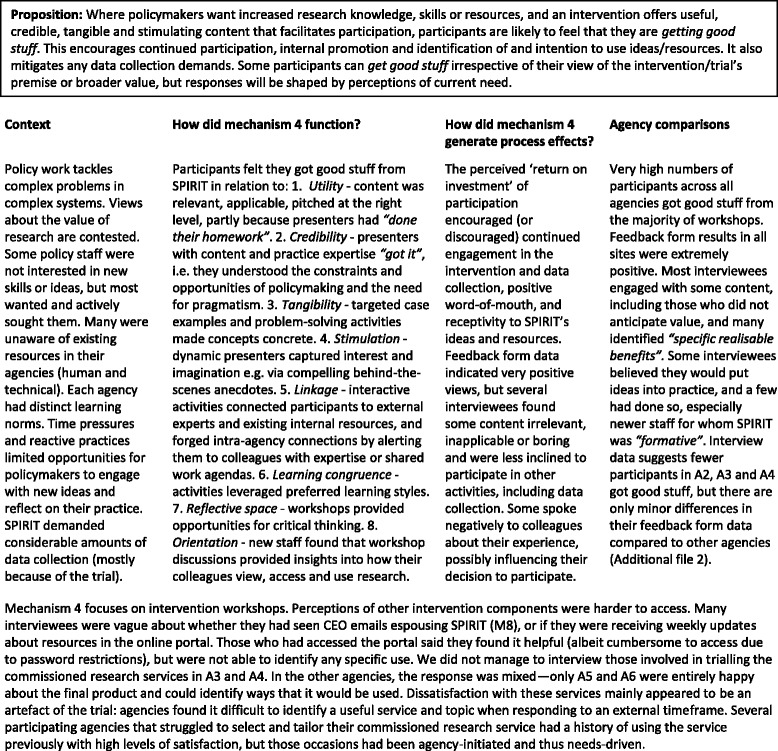
*Mechanism 4* – *“Getting good stuff”*

#### Mechanism 5

Self-efficacy (Table 6)

**Table 6:**
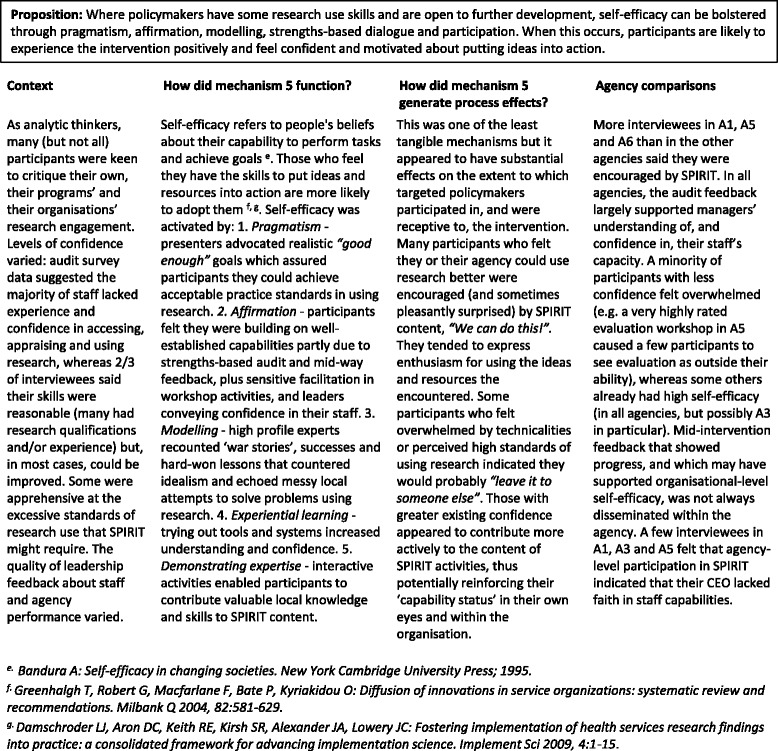
Mechanism 5 – Self-efficacy

#### Mechanism 6

Respect (Table 7)

**Table 7:**
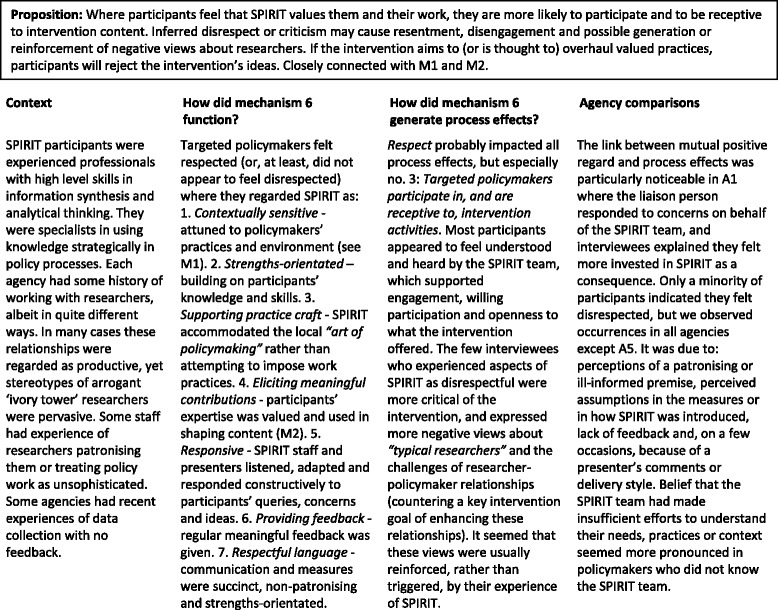
Mechanism 6 – Respect

#### Mechanism 7

Confidence (Table 8)

**Table 8:**
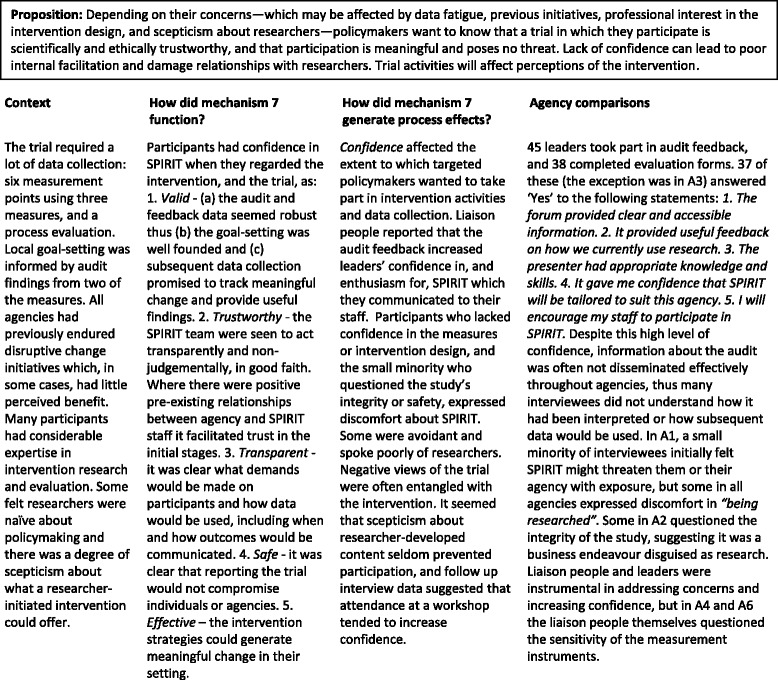
Mechanism 7 – Confidence

#### Mechanism 8

Persuasive leadership (Table 9)

**Table 9:**
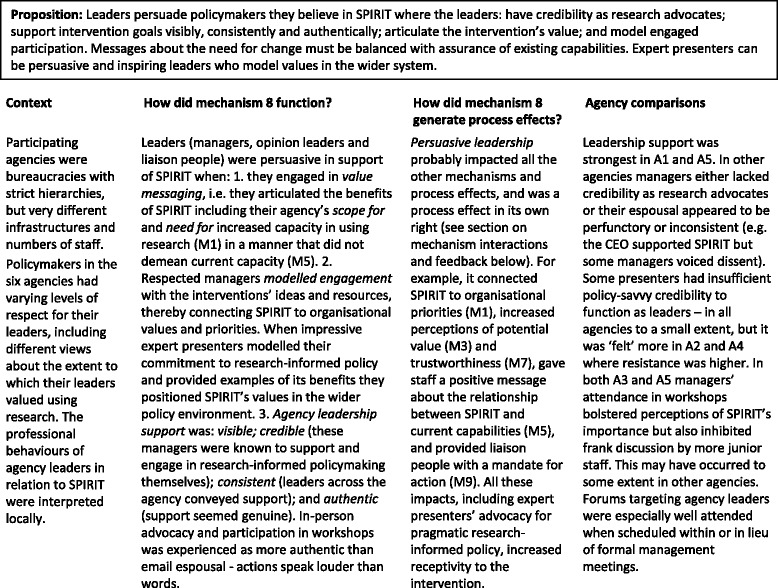
Mechanism 8 – Persuasive leadership

#### Mechanism 9

Strategic insider facilitation (Table 10)

**Table 10:**
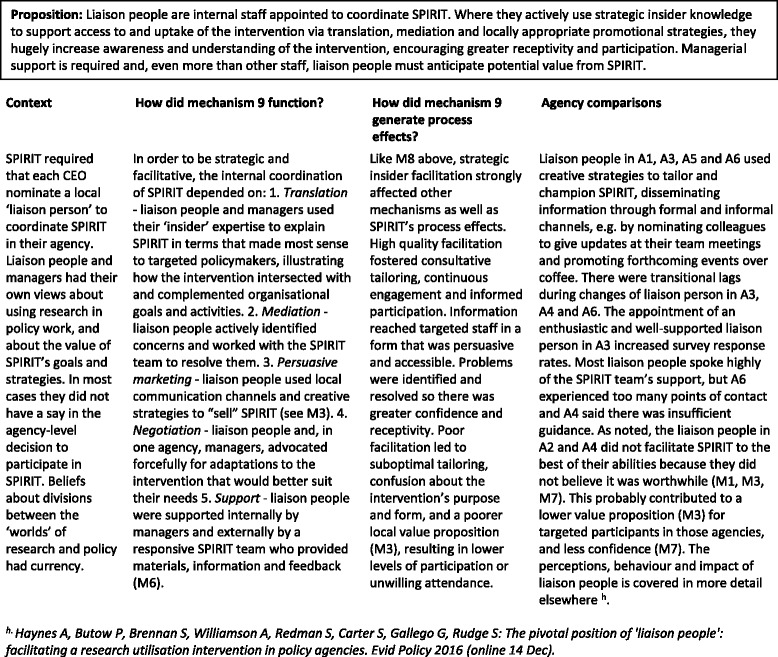
Mechanism 9 – Strategic insider facilitation

### Mechanism interactions and feedback

As others have noted, separating interactive processes into discrete mechanisms, while useful for theory building, fails to reflect their interdependence [61]. Many of the nine mechanisms include related concepts, which in some cases may be nested. For example, ‘self-determination’ (M2) is linked with ‘respect’ (M6) and may function as a mechanism within ‘self-efficacy’ (M5).

Figure 2 illustrates feedback within our model. This accords with the realist view that contexts, mechanisms and outcomes are not fixed entities but are contingent on the focus of the current evaluation, i.e. they function as a context, mechanism or outcome in a particular part of the analysis. Thus, many of our process effects feed back into and overlap functionally with the identified mechanisms, and may well function as mechanisms when this data is combined with the study outcomes. This is especially pertinent in a process evaluation given that process effects are hypothesised to mediate the intervention outcomes. An example of feedback is our finding that ‘persuasive leadership’ is a mechanism, despite one of the process effects being ‘Leaders support SPIRIT’. This is because we found ‘persuasive leadership’ to be crucial in activating other mechanisms (e.g. in asserting SPIRIT’s value proposition) and thus in achieving many of the other process effects.

We also concluded that mechanisms functioned on a continuum that encompassed negative and positive expressions. Mechanisms were activated to different extents in each agency and, on occasion, were activated negatively. For example, several interviewees made it clear that mechanisms such as ‘Self-determination’, ‘Getting good stuff’ and ‘Respect’ were activated negatively when they were instructed by their manager to attend a 2-hour workshop that had no relevance to their work

### Revised programme theory

These results enabled us to revise our programme theory to reflect contextual contingency, which also increases the operational transferability to other interventions and settings (Table 11).

**Table 11:** Initial and revised programme theory

Initial programme theory (a-contextual)	Revised programme theory (contextually contingent)
SPIRIT will engage and motivate agency leaders to ‘own’ the intervention using audit feedback, deliberative goal-setting and programme tailoring –this agency-driven approach will generate a priority-focused programme that offers locally relevant practice support and accommodates differences in agencies’ values, goals, resources and remits. The programme will comprise a suite of andragogical activities, tools and connections across the research-policy divide that provide resources and build knowledge, skills and relationships, and will be supported via modelling and opinion leadership by agency leaders and dynamic external experts. CEOs will promote SPIRIT in their agencies and liaison people will facilitate the tailoring and implementation – these strategies will act synergistically to stimulate and resource participants at different organisational levels, leading to changes in values, practice behaviours and agency processes. This will facilitate increased use of research in policy processes	Where agencies have an existing orientation to use academic research and are on a trajectory of improved use with perceived room for improvement, SPIRIT will be used to complement or trigger organisational initiatives. Where liaison people and agency leaders believe in the value of the intervention and have confidence in the measures, they will play a pivotal role in tailoring the intervention and championing its goals. Leaders will be motivated by deliberative audit feedback and goal-setting. In all sites, ownership will be increased by greater consultation, collaboration and choice. Agency-attuned communications will be vital in explaining goals, conveying value and addressing concerns. Andragogical activities, tools and connection across the research-policy divide will be valued in all agencies where they leverage existing strengths and address local concerns pragmatically. Staff will make use of these opportunities where they see concrete benefits, and newer staff may benefit most

## Discussion

From the participants’ perspective, the most positive attributes of the intervention were useful (i.e. relevant and applicable) content, high profile experts who delivered pragmatic content and demonstrably “*got it*”, active participation in intervention activities, and intervention flexibility supported by deliberative audit and feedback that informed goal-setting and customisation. Much of SPIRIT’s implementation fidelity was sound – all the components of the intervention were delivered – but activities were not always as interactive or as participant-driven as intended. Authentic in-person leadership support and committed liaison people were vital mediators, while obstacles included confusion about the purpose of participation in SPIRIT, perceptions of poor alignment with agency practices or priorities, and feeling misunderstood or judged. Previous organisational change initiatives and archetypal views of researcher-policymaker relations sometimes appeared to underpin expectations and frame some of the concerns. The data collection demanded by the stepped wedge evaluation was onerous, and aspects of the trial were often entangled with participants’ perceptions of the intervention. Like many others, we found that pre-existing positive relationships between the agency and those involved in designing and implementing the intervention had considerable facilitative effects [75–77]. In our case, they helped to activate mechanisms such as respect and confidence.

### Implications for intervention improvement

Given their pivotal importance, greater upfront engagement with each agency’s leadership and the nominated liaison person would have been beneficial. Local tailoring and shared decision-making was essential, but challenging for both the agency and the intervention team. For example, it was often difficult for agencies to make strategic use of processes that they had not initiated such as trialling the services for commissioning research. Advice from agencies about how tailoring could be best supported in their context may have been beneficial, but the process of tailoring will always demand time and effort. This reflects the underpinning need for agency leaders to be committed to participation from the start.

Despite being selected for broad similarities, the six participating agencies had markedly different remits, practices and conceptualisations of evidence. SPIRIT’s audit and feedback process was effective in developing a shared understanding of each agencies’ current and desired research use capabilities, but better understanding of their practice goals and values, and greater collaboration in designing the intervention and data collection instruments (which every agency desired) could have sharpened the meeting of minds about what was needed and how to address it. Understanding what participants think about intervention goals, and using their ideas about what should be done in order to achieve those goals, is usually critical for success [78].

As noted previously, the realist distinction between intervention activities and mechanisms is crucial for theory-driven evaluation, but it is equally crucial in the development of context-sensitive intervention design and implementation planning. An intervention cannot simply ‘do’ respect, or ‘deliver’ self-efficacy, it cannot control the perceived attractiveness of its premise, or make internal facilitators act strategically. Activating these mechanisms is an evolving work-in-progress shaped by personalities, relationships and complex shifting environmental opportunities and constraints. Greater understanding of the mechanisms that generate desired (and undesired) process effects provides helpful guidance, but putting this learning into practice takes creativity, humility and reflexivity.

### Our contribution

These findings add to the existing knowledge by surfacing evidence about how policymakers perceived and engaged with different aspects of an intervention trial designed to increase the extent to which they use research in their work. Our realist process evaluation approach goes beyond questions of implementation fidelity and ‘what works?’ to provide a more nuanced and theoretically informed account of how the intervention produced process effects, and why there was such variation across the six policy agencies.

As per Fig. 2, we anticipate that the intervention’s process effects, and the mechanisms that underpin them, mediate the study outcomes, but we caution against assumptions that this is a linear predictive relationship. As realist evaluation adherents indicate, there are usually multiple causal pathways in real world interventions, and the best we can do is identify common pathways for particular groups of individuals in particular circumstances; therefore, we concur with McMullen et al. that, “*there is not, nor can there ever be, a universal implementation model for complex interventions. Site-specific characteristics and realities need to be considered*” [79]. However, this consideration need not start from scratch with each new intervention – we can develop an increasingly sophisticated understanding of the conditions that make these outcomes more likely in a given setting. As Pawson argues, “*evaluation science assumes that there will be some pattern to success and failure across interventions, and that we can build a model to explain it*” [1]. We hope to have made a start in identifying these patterns in a form that will enable others to extrapolate and apply lessons to other interventions and contexts [1].

### Strengths and limitations of this process evaluation

Using a realist approach enabled us to identify and test hypothesised causal mechanisms, evaluate the extent to which SPIRIT activated them, use this analysis to refine the programme theory, and identify areas of strength and potential improvement in the intervention and trial design. The identification of underlying causal mechanisms and the development of propositions enhances the utility and transferability of the findings [3, 80] and strengthens the general knowledge base by building on existing theories. The thematic overview of the process evaluation data in Additional file 1, and the inclusion of informing theory in Additional file 2, provide ‘analytical trails’ that support the findings.

Triangulating different types of data obliged us to consider diverse points of view and increased the trustworthiness of our findings. As Wells et al. [9] note, “*… evaluations need to incorporate multiple methods, multiple sources and multiple perspectives if they are to reflect the context of practice adequately*”. We achieved this thanks to (1) the unusually generous appointment of a dedicated process evaluation researcher throughout the study, and (2) the length of the intervention (12 months) and its staggered delivery, which gave us considerable time in each agency to test hypotheses at different points in the intervention across six sites. However, we acknowledge this was an exploratory first step and the ideas are yet to be tested by others and in different settings; therefore, at this stage, our findings are only a rough indication of major causal patterns within SPIRIT’s engagement and participation. Further testing and refinement are required.

A limitation was our inability to determine the full range of views and experiences of targeted staff in each agency. Interviewees were sampled purposively for maximum variation of relevant views and experiences, but many declined interviews and it was not always possible to identify substitutes. Others have found similar problems [52]. Consequently, we reached a smaller range of participants than envisaged and so may have missed important views. For example, all the process evaluation interviewees in A4 (11 people with a total of 15 interviews over the duration of the intervention) were either lukewarm or dismissive of SPIRIT, but during outcome measures interviews some A4 participants stated that they welcomed the intervention, and following the trial their CEO said SPIRIT had impacted his agency positively. In all agencies, we saw some non-agreement between the highly positive feedback form data and the more critical responses in the interview data. This may be the result of different foci – interviews ranged across the whole of SPIRIT (including its premise, communication and data collection), while feedback forms were workshop-specific – but other factors could be skewed sampling, leading interview questions or the bluntness of the feedback form. The response rate for feedback forms was good, with 74% of attendees completing them, but it is unclear whether those who did not complete forms differed from those who did, and thus what views we might have missed. The direction of this quantitative data was consistent with patterns in the qualitative data regarding a more positive response from agencies 1, 5 and 6, but feedback form responses across agencies and items were so similar that it is likely that the tool discriminated poorly. We used Yes/No statements to maximise response rates from participants who might be rushing to leave, but this was probably too limiting. Certainly, there were many occasions where the free text fields conveyed ambivalence or, at least, scope for improvement, when the scored statements suggested 100% satisfaction. We would use a more sensitive instrument in the future.

### Reflections on conducting a realist process evaluation

Conducting a realist process evaluation was immensely valuable, but time consuming and challenging. Like others (e.g. [49, 81]), we struggled to disentangle aspects of the causal pathways; specifically, to delineate mechanisms from intervention strategies, contexts and outcomes. Realist analysis does not have a step-by-step guide, and it presents a unique tension between ontology and epistemology, so we sometimes struggled to reconcile our search for factual existing mechanisms with the need to take an “*imaginative leap*” and postulate those mechanisms [82]. Three strategies helped: first, scanning appropriate literature and drawing on established theories, for example, the concept of relative advantage [6, 58, 83] was critical for understanding variation in perceptions of SPIRIT and how this linked to the communication strategy. Second, the realist emphasis on counterfactual thinking [54] was very helpful in weighing up the plausibility of different theories. Third, reminding ourselves that causality does not function as discrete components or configurations and that our analysis was intentionally abstracting for the purposes of theory building rather than attempting to depict reality in all its messy, interdependent glory (see also [61]).

## Conclusion

This realist process evaluation describes how participants experienced different aspects of a multi-component research utilisation intervention in policy organisations, and why there was such variation across the six implementation sites. We identify nine mechanisms that appeared to facilitate engagement with and participation in the intervention in these settings: (1) Accepting the premise (agreeing with the study’s assumptions), (2) Self-determination (participative choice), (3) The value proposition (seeing potential gain), (4) ‘Getting good stuff’ (identifying useful ideas, resources or connections), (5) Self-efficacy (believing ‘we can do this!’), (6) Respect (feeling that SPIRIT understands and values one’s work), (7) Confidence (believing in the study’s integrity and validity), (8) Persuasive leadership (authentic and compelling managerial advocacy) and (9) Strategic insider facilitation (local translation and mediation). This analysis was used to develop tentative propositions and to revise the overarching programme theory. Although our findings are nascent and require further testing and refinement, they indicate areas of strength and weaknesses that can guide the development and implementation of similar studies in other settings, increasing their sensitivity to the range of issues that affect the value and compatibility of interventions in policy agencies.

## Additional files

Additional file 1: Descriptive overview of results. (PDF 403 kb)

Additional file 2: Supporting theory. (PDF 335 kb)

Additional file 3: Summary of SPIRIT intervention implementation fidelity. (PDF 1460 kb)

## Abbreviations

CEO: Chief executive officer
SPIRIT: Supporting Policy In health with Research: an Intervention Trial
