# Supplementary material for: Policymakers’ experience of a capacity-building intervention designed to increase their use of research: a realist process evaluation
Source: Health Res Policy Syst. 2017 Nov 23;15:99. doi: 10.1186/s12961-017-0234-4 (PMC5701502; doi:10.1186/s12961-017-0234-4)
Supplement: Supplementary file 1 — Descriptive overview of results. (PDF 403 kb) [file 12961_2017_234_MOESM1_ESM.pdf]

Additional file 1. Descriptive overview of results

Mechanism 1. Accepting the Premise

To be perceived as relevant and potentially beneficial, SPIRIT had to establish its compatibility with each agency's remit, practices and current trajectory of change. In half the agencies the intervention was regarded as a good fit that completed existing or planned initiatives—*"it reinforced what we were trying to do anyway"*—and many interviewees saw SPIRIT as addressing *"a real need"*. This translated into some enthusiasm for the intervention, particularly among groups of participants in A1 and A5 who were looking for ways to improve the use of research in their practice. In two agencies the liaison people and many of the other participants rejected the premise of SPIRIT because there was a perceived disconnect between the intervention's assumptions and the agency's values; specifically, differences in their conceptualisations of evidence and how it should be developed (A4), and differences in their beliefs about how interventions should be designed and implemented, which amounted to feeling that SPIRIT was imposed and too prescriptive (A2). The agencies' values were bound to the needs and preferences of their primary stakeholders, which for A2 and A4 meant that clinicians' views and 'bottom-up' initiatives had primacy. A3 struggled to accept the premise of SPIRIT for different reasons. They self-identified as having such high standards of research use that, according to one manager, there was *"no room for improvement"*. This view was echoed by other staff who were surprised their CEO felt participation in a research utilisation intervention was warranted.

Even where they saw substantial scope for improvement, some staff in all sites questioned the need to address their use of research as a priority, *"I don't think there's a need for any kind of urgent remedial action here"*. In A2, some interviewees argued that they should not have to improve their ability to access and appraise research, believing that research experts should be doing this work, *"why can't they just do it for me rather than tell me how to do it?"*. One interviewee admitted she was not using research in her work (despite plenty of scope and the availability of relevant studies), yet rejected the need for SPIRIT because *"We're doing it already"*. There was a sense that their use of research was good enough for their purposes, and that they would prioritise other issues if they wished to improve practice.

Surprisingly, *Accepting the Premise* was not a universal deal breaker. It had profound impacts on liaison people (see below), but many other participants who rejected the premise of SPIRIT appeared to assess each intervention activity on its own merits—possibly because they were unaware that they were part of SPIRIT—and often reported that they found workshops worthwhile. We do not know if, or how many, potential participants failed to take part because they had dismissed the intervention as incapable of benefitting them or their agency.

This mechanism seemed to have a more fundamental impact on liaison people who were required not only to administer the study but to attach themselves to it as champions. In the two agencies where they rejected the premise it profoundly influenced how they tailored, administered and promoted SPIRIT (M9). Rejecting the premise also appeared to reinforce beliefs about a generalised disconnect between researchers and policymakers, and to fuel scepticism about the agenda of research-informed policymaking, *"I just think it probably just reinforced to me that what we do is a lot more complex than perhaps how academics can describe it."* Acceptance of SPIRIT's premise was bolstered where there was advocacy support from management (M8) and strategic internal facilitation (M9). The deliberative conversations held with managers as part of the audit feedback galvanised leadership support and helped to calibrate the intervention with each agency's needs, but was not always sufficient to address fundamental concerns about the underlying premise.

Mechanism 2. Self-determination

This mechanism incorporates interactions that enabled participants to regard SPIRIT as empowering and self-driven rather than constraining and imposed. Self-determination was a process as well as a destination, and is strongly linked to M6: Respect.

As intended, the intervention's value was undoubtedly enhanced by its flexibility, enabling agencies to identify local goals and tailor content for increased relevance and applicability. Policymakers naturally want to be treated as experts in their domain, and the audit feedback forums were highly successful in recognising and building on this. This contributed to a sense of ownership in four agencies but, despite positivity about the audit feedback deliberations (which were very well received in all agencies), some leaders in A2 and A4 argued there was insufficient flexibility to use SPIRIT in a way that suited them, and that fixed options contributed to poor compatibility. Given that the form of SPIRIT was not flexible enough for its premise to be customised, tailoring was only embraced in agencies where the exiting premise was acceptable (M1). Tailoring was also onerous: agencies often struggled to match SPIRIT options to their needs, and decision-making was complicated by bureaucratic processes so that some liaison people had to develop new pathways for making and authorising decisions about customisation. Liaison people and managers put very different levels of effort into this consultation and customisation, but where managers and, particularly, liaison people actively engaged in tailoring, they shaped SPIRIT more than anticipated. For example, A6 insisted on greater customisation than was initially allowed, A5 negotiated 'extras', and A3 modified the participant eligibility in their setting. Where liaison people consulted widely, interviewees generally saw the intervention as more attuned to their needs.

Opportunities for self-determination affected how people engaged in and perceived workshops. The expert presenters were asked to make workshops highly participative, but interactivity varied hugely. At its best, participant contributions shaped the content via questions, case examples and robust debate. At worst, participants felt *"lectured at"* (which also negatively triggered M6: Respect). Although they were encouraged to co-present workshops, only one agency took this up. This workshop was very well received, but since most of the workshops were very well received and the feedback form instrument discriminated poorly, it is not possible to determine how much difference co-presentation made.

A few interviewees, particularly in A1 (which was most involved in policymaking rather than program development) worried that SPIRIT was critical of 'craft' practices and might assert a protocol dictating how research should be used that would *"infringe on the art of writing policy"*. They wanted the freedom to use research flexibly, as they saw fit. Some participants felt obliged to attend workshops and complete measures due to managerial expectations or, in one agency, explicit demands. A few liaison people took on the role reluctantly, but this probably only affected implementation in A2 and A4 as the other 'put upon' liaison people were not involved in the active phase of SPIRIT.

Trial demands surrounding the intervention affected self-determination. The intervention's randomised start date was inconvenient for some agencies (so much so that one agency insisted on a postponement). In several sites, participants felt their agency could have used the intervention more strategically if they had been able to determine when it took place. Most interviewees would have liked the whole-of-study to have been collaborative, arguing that the intervention form and content, and the data collection instruments, would have been better attuned to their agency as a consequence.

Mechanism 3. The Value Proposition

A *"value proposition"* (promised advantage) is a convincing argument about the worth of a strategy that is assessed by prospective users on the basis of perceived costs and benefits [1]. In the case of SPIRIT, the intervention as a whole and its individual activities were judged on their value proposition. This judgement affected receptivity to the intervention's ideas. Most participants saw potential value in SPIRIT but were *"agnostic"* prior to the intervention's start. They invested time based on anticipated utility—*"concrete payoff"*— and, to a lesser extent, interest. They were attracted by high profile presenters with *"big names"* and impressive biographies; persuasive promotion of SPIRIT activities by managers and the liaison person; and knowing that content had been tailored for them. The mix of presenters from research and policy fields was valued by most, but some had low expectations of researchers, *"I just think, oh, academics, we're going to get talked at and get a lot of overcomplicated things, not in plain English"*. Value was often deferred, many individuals commented that they had little to gain from SPIRIT themselves, but that others (more junior or less experienced colleagues, or the agency's executive) could benefit.

Communications were crucial in establishing the value proposition. Participants in all agencies wanted five questions answered: *1. What's in it for us? 2. What do we have to do and when? 3. Why are we doing it? 4. Who are you?* and *5. What are you doing?* However, it took some time before the SPIRIT team caught up with this. Early communication was poor, causing confusion and some alienation. SPIRIT's acronyms were universally loathed. Some participants' beliefs that researchers and policymakers operate on different planes were exacerbated by dense, jargon-laden information. Consequently, many participants were unsure why their agency was participating in SPIRIT, *"there is a bit of confusion… 'What are we doing this for?'"*. In A1, managers used a key phrase from a workshop—*"using the best available research in the time available"*— to encapsulate their goals in participating *and* their philosophical stance in relation to research more generally. But other agencies struggled to identify exactly what they hoped to get out of SPIRIT. Ultimately, persuasive marketing was dependent on acceptance of SPIRIT's premise (M1).

Communications were also critical in minimising the threat that trial demands posed to the value proposition. For example, poor understanding of SPIRIT undermined confidence (M7): the six measurement points had been explained but remained puzzling for many, partly because of the non-intuitive rationale for multiple measurement points (unlike before and after measures). This led to *"survey fatigue"* and a perception of redundancy, *"I wonder why we had to have two baselines?"*. Some participants thought data collection interviews were part of the intervention. Where data collection was framed by liaison people as part of the value proposition (in that it by provided performance indicators or operated as part of SPIRIT's quid pro quo), response rates increased. Where agencies had multiple data collection points prior to the intervention (because they had been randomised to a later start date) , holding strategies were required to emphasise forthcoming returns on investment.

Mechanism 4. "Getting Good Stuff"

Feedback form data indicated that workshops were extremely well received (Table 1). Across all agencies, almost all participants judged that the workshops were interesting, and had presenters with appropriate knowledge and skills, and reported that SPIRIT was likely to be beneficial to their agency (items 1, 4 and 6: ≥ 96% of responses across all agencies). A slightly lower, but still high, proportion of responses indicated that participants expected to use information from the workshops in their work (item 5: ≥ 93% of responses across all agencies). Perceptions of the workshop's relevance (item 2) and the extent to which it was realistic about the challenges and constraints of the agencies work (item 3), were the only items where any potentially important differences between agencies were observed. Perceptions of relevance were marginally lower in A2 compared to the other agencies (followed by A4), and the extent to which the workshops were regarded as realistic about the agency's work were marginally lower in A4, followed by A3.

Table 1. 'Yes' responses on SPIRIT workshop feedback forms: total numbers and percentages

Feedback form statement	A1	A2	A3	A4	A5	A6	All agencies
1. The workshop was interesting	77 (100%)	92 (97%)	82 (98%)	66 (96%)	103 (98%)	71 (100%)	491 (98%)
2. The workshop was relevant to my work	73 (95%)	86 (90%)	81 (96%)	63 (91%)	97 (92%)	72 (100%)	472 (94%)
3. The workshop was realistic about the challenges and constraints of our work	41 (100%)	42 (93%)	23 (89%)	35 (83%)	65 (94%)	56 (98%)	262 (94%)
4. The presenter had appropriate knowledge and skills	83 (100%)	101 (100%)	90 (98%)	75 (96%)	110 (99%)	76 (99%)	535 (99%)
5. It is likely that I will use information from this workshop in my work	41 (98%)	32 (94%)	67 (93%)	53 (96%)	81 (96%)	51 (94%)	325 (95%)
6. It is likely that SPIRIT will benefit my agency	33 (100%)	24 (100%)	57 (100%)	46 (96%)	74 (99%)	46 (96%)	280 (98%)

We observed small differences in the mean total feedback score across agencies, with the lowest overall scores reported in Agencies 2 and 4 (Figure 1). The maximum difference between any two agencies was 1.4 (out of 6), or the equivalent of a different response to one of six items. Agency 2 had lower score than all other agencies (ranging from 0.9 lower than agency 4 to 1.4 lower than agency 5). The difference in the mean total feedback score between other agencies was small (from 0.2 to 0.6 out of 6).

Figure 1. Total feedback form scores for each agency in relation to the six statements

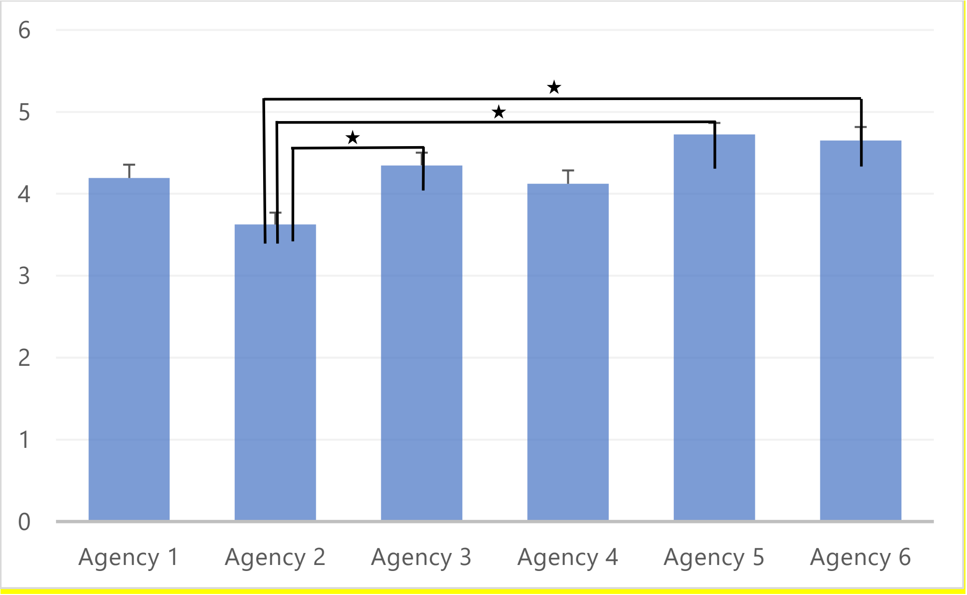

** Significant differences are marked with a star*

Many interviewees concurred with the positive feedback form responses, "*They were some of the best interventions I've ever seen*". The use of passionate *"service orientated"* presenters with hands-on expertise who had *"done their homework"* and who *"got it"*, together with compatibility with preferred learning styles, resulted in useful and *"invigorating"* content that participants hoped to apply. Targeted case examples and memorable insights provided by behind-the-scenes stories, especially those recounting hard won lessons, were particularly valued. The opportunity to talk with researchers was appreciated when the topic was regarded as relevant and applicable, and four agencies planned further seminars or collaborations with a presenter. Although many interviewees felt their learning had been consolidated rather than advanced, this was usually seen as worthwhile. In agencies with unengaged liaison people (A2 and A4), interviewees viewed SPIRIT as having less value overall, but, despite rejecting the premise (M1), and in A2 feeling obliged to attend (M2), many participants reported that they found individual workshops useful.

Policymakers in all agencies valued the opportunity for reflection and to explore topics with colleagues across the agency—*"It really brought people together"*—which sometimes led to the identification of shared interests and internal resources. Newer members of staff used workshops as orientation, a chance to gauge alignment between agency norms and SPIRIT's idea, while those without research expertise were most enthusiastic about SPIRIT providing valuable ideas and tools, *"[SPIRIT happened] at the very start of me working here, so it's been formative for me"*. In A3, where staff saw little room for improvement, SPIRIT affirmed their view, *"So we're now walking around very conceited, with very big heads, thinking... we use evidence in the best way possible... [but] it's nice to have it confirmed"*. Several managers and liaison people identified value in forging or enhancing relationships with the SPIRIT team, a side effect of participation.

Where workshops failed to align with local learning practices (e.g. not distributing advance readings) they were regarded as having missed an opportunity and were considered less useful (A2 primarily). As anticipated, didacticism was unwelcome, but interactivity was hard to assert as some presenters did not follow their brief, and liaison people sometimes attempted to increase the value of workshops by

maximising content at the expense of participation, which often appeared to have the opposite effect – participants felt overwhelmed and unable to take in key messages.

Perceptions of other intervention components were harder to access. Many interviewees were vague about whether they had seen CEO emails espousing SPIRIT (M8), or if they were receiving weekly updates about resources in the online portal. Those that had accessed the portal said they found it helpful (albeit cumbersome to access due to the need for a password), but could not identify any specific use. We did not manage to interview those involved in the brokered services in A3 and A4. In the other agencies, the response was mixed—only A5 and A6 were entirely happy about the final product and could identify ways that it would be used. Dissatisfaction mainly appeared to be an artefact of the trial: agencies found it difficult to identify which service and what topic would best meet their needs when required to do so within an externally imposed timeframe. Several participating agencies that struggled to select and tailor their brokered service had a history of using the service previously with high levels of satisfaction, but those occasions had been agency-initiated and thus needs-driven.

The six data collection points demanded by the trial tainted some participants' view of the intervention's value, but was usually judged to be "*worth the hassle*" due to the value of free high calibre workshops and other intervention resources.

Mechanism 5. Self-efficacy

Two thirds of those who contributed to the process evaluation said they used research on a regular basis and had a reasonable degree of existing capacity, albeit with room for improvement in most cases. Some felt they had the capacity but that their current role or project did not require research input. A few felt they neglected opportunities to use research either because they lacked the skills and confidence, or because of time pressures and insufficient onus on research use in the organisational culture to warrant prioritising its use, *"[we] don't have time to sit around and ponder... [we have] to get on and do something*".

Some participants reported that SPIRIT workshops had raised the bar in terms of research appraisal or program evaluation, but in agencies where evidence was seen as more fluid and consensual (or driven by *"political imperatives"*) there was little perceived need to improve their mastery of academic research utilisation. An example of this was the emphasis on stakeholder advice as core evidence in A2 and A4, *"I think the culture is you just get up and go to the person that's in our network and say 'I've been asked to do something on this. Who shall I talk to or can you head me in the right direction?'"*.

Nevertheless, participants identified several intervention strategies that they believed supported self-efficacy generally. Where workshops were explicitly pragmatic it gave permission for *"good enough"* practices that participants found motivating, *"We can do this"*. Many mentioned the message *"Do the best you can with what you can get in the time available"* (from the Leaders' Forum run by Prof John Lavis) as especially helpful. As one of the liaison people put it *"the last Leaders' Forum really galvanised people... it was practical, realistic.... it was potentially technically challenging to get it in place but something that could be done."* Another participant talked about her confidence that she would be able to use resources provided by SPIRIT, *"it gave the idea that once you've identified them implementing them actually isn't that complicated. It's sort of using the same skills that you're already using... so you don't need to have a PhD to be able to understand research and think about how you might apply it to your work."* Presenters' forthright accounts of their struggles and successes in using research in policy acknowledged real world challenges and provided realistic goals. Participants valued opportunities to contribute expertise via discussion and, in one case, co-presenting. They particularly appreciated it when providers' recognised that the agency was already research-engaged and had developed skills in integrating research with other forms of locally-relevant evidence. Workshops were also valued as a rare chance for critical reflection, *"space to think about research and where it fits in"*.

Affirmation by presenters, and via the audit feedback, showed agencies that they were building on well-established capabilities and were already part of the way there, *"It was confirmation we're heading in the right direction"*. However, it was not possible to pitch workshops at the right level for all members of heterogeneous staff groups, so there were winners and losers. Some participants gained nothing new and,

in some cases, those with little expertise felt overwhelmed. For instance, after an evaluation workshop an epidemiologist explained, *"if you felt confident in [evaluation] that would be misplaced confidence.... That's what I took from that."* Affirmation by leaders also bolstered confidence, but a few interviewees (mostly experienced, mid-level managers) inferred that agency participation in SPIRIT meant their CEO believed staff lacked competency in using research.

The SPIRIT team anticipated that the brokered services would provide useful experiential learning in commissioning research and evaluation, but most interviewees who were involved in that component of the intervention were already experienced in commissioning and did not find the process itself to be particularly valuable.

Mechanism 6. Respect

This was a fundamental mechanism that was anticipated by the designers and was successfully incorporated in most of SPIRIT's activities. However, it had a profound effect when it was activated negatively, which the context made more likely: many participants evoked pre-existing views about *"typical researchers"* being both naïve and arrogant. Even if they did not subscribe to these views—in fact, many reported positive experiences of working with researchers—it was a pervasive cultural stereotype. In some cases it may have contributed to a conscious decrease in contact with researchers, for example, an executive in one agency explained that they had found university based researchers to be *"so far from reality"* that the organisation tended to *"stay away from academics more often than not"*.

The extent to which SPIRIT was perceived as an attempt to infringe on the *"art of policymaking"* (discussed in *Mechanism 2. Self-determination*, above) was also seen as an indicator of respect. Experienced policymakers wanted recognition of their expertise.

Perceived lack of respect in aspects of SPIRIT caused resentment and, in some cases, withdrawal. Participants reported the following triggers: presenters making assumptions about or downplaying the challenges of participants' work; presenters failing to elicit participants' input in workshops; failure by SPIRIT staff to be responsive or ensure participants were *"kept in the loop"* via regular feedback; feeling judged when being interviewed for the measures (in two agencies); patronising language and the perceived assumption that policy staff had poor understanding of research or needed to engage with research differently (some examples in all agencies); and the lack of options re shaping SPIRIT. In every agency, some participants felt SPIRIT had a deficit approach and called for a more inductive needs analysis that built explicitly on their practice strengths, but this was particularly pronounced in A2 and A4.

Mechanism 7. Confidence

Most participants, particularly in A1, A3, A5 and A6, expressed or implied confidence in SPIRIT, particularly where they had positive views of, or existing relationships with, the SPIRIT team, but some were sceptical about the sensitivity of the measurement instruments. This included two liaison people (who were responsible for facilitating data collection): one doubted the measures captured meaningful information about their practice, and the other felt they did not align with behaviours targeted by the intervention. For a few participants, these concerns undermined confidence in using audit findings to inform goal-setting. However, interviews with agency leaders and the liaison people clearly showed that the deliberative conduct of the feedback forum itself boosted confidence in the intervention. Evaluation forms supported this. 37 of the 38 leaders who completed evaluation forms (the exception was in A3) answered 'Yes' to these statements: *1. The forum provided clear and accessible information. 2. It provided useful feedback on how we currently use research. 3. The presenter had appropriate knowledge and skills. 4. It gave me confidence that SPIRIT will be tailored to suit this agency. 5. I will encourage my staff to participate in SPIRIT.* But information about the audit was often not always disseminated effectively, thus staff at other levels of the organisations sometimes did not understand the findings or how they would be used.

A number of interviewees expressed scepticism about SPIRIT's ability to effect change. They argued that engagement with ideas and resources via two-hour workshops— no matter how inspiring the workshops were—was not enough to alter established practice. Two limitations were identified. First, SPIRIT's limited

ability to harness learning mechanisms with a predominantly workshop-focused intervention; the intervention was providing *"refreshers"* rather than training. Second, they pointed to organisational culture and current practice norms as powerful counter agents, and argued that change mechanisms had to be *"embedded in our day-to-day processes"* if practices were to change and be sustained. They recognised the role of organisational systems and leadership in making this happen.

A minority of interviewees across all sites expressed suspicion that data about individuals' or teams' performance might be used internally. This concern appeared to be sparked by experience of previous interventions and was more pronounced in agencies with less engaged liaison people, apparently because liaison people were actively addressing concerns in other agencies. Conducting the intervention as part of a trial added another layer of threat regarding potential public exposure; for example, the danger that publication of sensitive evaluation data might show the agency in a poor light, possibly by comparing it with other agencies participating in the trial.

A small minority of interviewees questioned the agenda behind the trial, *"who will ultimately benefit?"*, while some in A2 openly questioned the integrity of the study, *"are you actually just gunning for business?"*. Such concerns may have been exacerbated by the increasingly market-orientated culture that the agencies were operating in, and their own desire to maximise returns on investment from commissioned and partnered research rather than simply handing out funds.

Initially, some interviewees doubted researchers could develop policy-useful content, but participation in intervention workshops seemed to diminish this belief. Some participants expressed discomfort at *"being researched"* and a few blamed this on their agency's CEO, *"She [the CEO] determined she wanted to put us in a petri dish and compare us to others"*.

Most concerns were satisfactorily addressed where liaison people worked with the SPIRIT team to actively identify and respond to them (M9), and authentic leadership support appeared to bolster participants' confidence in SPIRIT overall (M8).

Mechanism 8. Persuasive leadership

Interviewees in all agencies reported that some managers encouraged participation in SPIRIT, but this was especially consistent in A1 and A5. Support was perceived as most genuine, and most memorable, when managers promoted SPIRIT informally and in-person. For example, interviewees usually did not recall the quarterly email endorsements from their CEO, or regarded them as corporate diplomacy, but they were impressed when senior managers made ad hoc expressions of support in conversations and meetings and, particularly, when they attended workshops: *"seeing our directors engaged has meant that we're seeing [SPIRIT] as more important"*. Managers' comments during activities also provided guidance about how to relate to the content, including what ideas were important and how they applied to agency practice. High profile external experts—known leaders in their field—modelled a broader commitment to research-informed policy.

Aware that their participation could increase the value of SPRIT, some managers in most agencies, including CEOs, attended workshops to model engagement (*"set a good example"*). However, in A3 only one executive member participated, and only briefly. In the largest and most distributed agency, A6, workshop attendance was more strictly delineated so frontline staff were largely unaware that executive managers were participating in SPIRIT activities. Managers and the liaison person in one site undermined their CEO's authentic endorsement of SPIRIT by showing distain for the intervention's goals and form. Thus visible leadership support for SPIRIT did increase perceptions of its value, but mostly where this support was consistent across the management team, in-person and demonstrably genuine. A downside was that, on occasion, managers' presence in workshop inhibited frank debate, particularly where discussion focused on current weaknesses in research use.

Mechanism 9. Strategic Insider Facilitation

Liaison people and some leaders played a critical role in improving participants' grasp of SPIRIT, using their insider knowledge to translate and maximise uptake of information. But the extent to which the liaison people accepted the premise of SPIRIT (M1) and were thus able to function as genuine champions varied considerably. Crudely speaking, in four agencies they championed SPIRIT and in two they did not. In agencies with less engaged liaison people, interviewees viewed SPIRIT as having less value overall, some staff did not know about SPIRIT activities and many were unaware that the intervention had been tailored for them. Conversely, where liaison people were most active they used creative strategies to promote upcoming activities and disseminate information through formal and informal channels, e.g. by nominating people to give updates at team meetings and encourage participation by their peers. And they functioned as mediators, ensuring that misconceptions and concerns were identified and addressed, and the SPIRIT team were advised of problems as they arose. Research team responsivity (see M6) was also vital for successful mediation. For example, liaison people could resolve complaints about the length of the online survey because the research team shortened it. The perceptions, behaviours and impacts of liaison people are explored in a dedicate paper published previously [2].

References for Additional file 1.

1. Barnes C, Blake H, & Pinder D (2009) *Creating and delivering your value proposition: managing customer experience for profit* Kogan Page Publishers.
2. Haynes A, Butow P, Brennan S*, et al.* (2016) The pivotal position of 'liaison people': facilitating a research utilisation intervention in policy agencies. *Evidence & Policy* https://doi.org/10.1332/174426416X14817284217163.
