# Supplementary material for: Policymakers’ experience of a capacity-building intervention designed to increase their use of research: a realist process evaluation
Source: Health Res Policy Syst. 2017 Nov 23;15:99. doi: 10.1186/s12961-017-0234-4 (PMC5701502; doi:10.1186/s12961-017-0234-4)
Supplement: Supplementary file 2 — Supporting theory. (PDF 335 kb) [file 12961_2017_234_MOESM2_ESM.pdf]

Additional file 2. Supporting theory

Mechanism 1. Accepting the premise

To succeed, interventions (including research utilisation interventions [1]) have to address a real need , i.e. to offer greater benefits than current practice [2-4]. To do so they must be perceived as compatible with participants' and organisation-wide' beliefs, assumptions, values and work practices [3, 5] and tackle a pressing concern [6]. Staff will resist an intervention if it does not appear to be in the interests of their agency or its primary stakeholders [7], or if there is a belief that *"we already do that"* [8]. Organisational characteristics (e.g. absorptive capacity and receptivity to change [9]) affect how staff value evidence [1], and different conceptualisations of evidence will be meaningful in different contexts, e.g. 'social proof' (knowing through doing) may be more compelling than 'scientific proof' [10]. Policymakers do not always trust the institution of research or find its outputs legitimate [11]. Audit and feedback theory posits that performance data which shows a gap between current and desired practice can establish the need, and create motivation for, change [12]. This requires credible audit data (M7) and shared goals.

Mechanism 2. Self-determination

Self-determination is the feeling of having control [13]. The value of involving participants in the design and implementation of interventions is well established – top down interventions seldom work [3, 14-16]. Eliciting participants' views about the legitimacy of intervention goals, and the methods for achieving them, is critical for success goals [17]. New ideas and resources are more likely to be taken up if those they target *"have sufficient opportunity, autonomy, and support to adapt and refine the innovation to improve its fitness for purpose"* [3]. Adult learners want to self-direct their professional development and draw on experiential knowledge [18], so workshops must be flexible and participative. Where possible, participants should be involved in planning their learning. [4]

System-wide trends can encourage uptake of interventions [2] but if staff may feel obliged to take part [19] this can result in symbolic participation with minimal follow-through. There is an important difference between 'towing the line' and 'deep engagement' [7]. Incentives can motivate participation, provided they are targeted appropriately [20]. Greater self-determination is positively associated with motivation to change [21] and ownership of change processes. [10, 22]

Mechanism 3. The Value Proposition

It is critical that participants are aware of and understand the intervention and its goals, and how intervention activities are likely to affect them [2, 3]; but multi-component interventions may be harder to understand [23]. Policymakers want clear, succinct information, yet researchers often default to jargon [24]. Participants will make judgements about the intrinsic value of the intervention based on how *"the intervention is bundled, presented, and assembled"* [2]. This *"selling game"* requires insider rhetoric and leadership support. [8]

As Karanika and Biron (2013) argue, *"change, be it at the individual, organizational or societal levels, is about individuals making sense of and assigning meaning to events and their environment"* [25:241]. This meaning, while made internally, is developed through comparison and negotiated with peers [25, 26]. It can be reframed, but it cannot be managed in a wholly predictable manner [3] - ideas travel through organisations in fluid ways [27]. The question of 'What's in it for me?' and the ways that individuals express their views about value will affect peer enthusiasm or resistance [2] and overall engagement. [14]

Mechanism 4. "Getting good stuff"

Relevance and usability are recognised as critical features in intervention science [3], adult learning [4] and research utilisation [24]. As adult learners, policymakers are problem-centred and want practical ideas that have clear applicability [4]. They benefit from being involved in the content development [4, 20, 28]. Workplace learning benefits when presenters understand the learner in context and use appropriate

narratives [29]. Vivid and detailed content increases learning, as does modeling struggle rather than effortless success [30]. Critical reflection—a well-established strength in individual and organisational learning— is positively associated with participation and self-efficacy (M5) [31]. Data collection burdens can decrease willingness to participate overall. [32]

Mechanism 5. Self-efficacy

Self-efficacy refers to people's beliefs about their capability to perform tasks and achieve goals [28]. Those who feel they have the skills to put ideas and resources into action are more likely to adopt them [2, 33], and more likely to welcome and commit to organisational change initiatives [34]. Thus self-efficacy increases motivation and persistence, but can also function as a vicious circle of self-doubt [20, 35].

Interventions are more likely to build self-efficacy when they identify strengths, use credible experts to model values and behaviours, and enable participants to practice new skills [28, 35]. Participants should feel they are knowledgeable and valued partners in the learning process, and have a safe space for reflection (M7) [2, 4]. Leaders play an important role in building self-efficacy via showing confidence in their staff and providing encouraging feedback [35]. Feedback can be used to create a sense of 'wins' in relation to intervention goals [6]. Existing beliefs about self- and organisational-efficacy affect the extent to which participants are willing to acknowledge scope for improvement and feel capable of owning the intervention goals - *"If people cannot take care of a problem, they won't see a problem"*.[10:8]

Mechanism 6. Respect

Engagement and appreciation is a two-way process, participants need to feel valued in order to value in return [36]. Other studies emphasise the need for mutual respect in researchers/policymaker interactions [37] yet researcher/policymakers relationships are all too often characterised by separation, mistrust and poor understanding [38, 39]. Archetypal stereotypes of researchers and policymakers continue to have currency. [40]

Strengths-based practice advocated in social work and education, and more recently in public health, emphasises the importance of respecting participants via inclusion in intervention design and recognition of capabilities [41], while adult learning, social cognitive theory, audit and feedback theory and implementation science all emphasise the importance of participation and feedback on engagement [2, 18, 28, 42].

Much of the more sophisticated research utilisation literature describes policymaking in terms of craft in which research is adapted rather than adopted [e.g. 24, 43], and implementation science makes a similar point in relation to interventions overall – they must be flexible enough to accommodate effective local practices. [e.g. 2, 3, 44, 45]

Disagreements can provide a platform for demonstrating goodwill and commitment to the relationship [46]. Efforts made by researchers to resolve concerns may increase the perception of being respected and instil greater confidence in their intentions.

Mechanism 7. Confidence

Frontline staff want initiatives to be credible and trustworthy, i.e. to have a strong evidence-base but not threaten professional autonomy e.g. [27]. Thus potential participants weigh up *"the quality and validity of evidence supporting the belief that the intervention will have desired outcomes"* [2]. The source (internal or external) and perceived legitimacy of an intervention affect how people engage with it [47]. People are less likely to embrace an intervention where it carries risks [3]. This also applies to internal facilitators (M9) who may feel they are taking risks in championing the intervention [48]. Financial costs can affect attitudes towards an intervention [2] but it is not known how receiving free content affects confidence or perceptions of an intervention's value in policy settings.

People who have endured multiple workplace change initiatives with little perceived benefit become suspicious and pessimistic [6]. Indeed, scepticism can be a major barrier to engagement, and often requires specific strategies to address it [16]. Attending to the history of an organisation is critical when developing appropriate drivers for future change [49]. Leaders can influence staff confidence, but may be dismissed if the intervention is seen as a top-down managerial initiative that is out-of-touch with on the ground realities, e.g. [10, 27].

The efficacy of audit and feedback is dependent on the perceived legitimacy of the data that informs it [50]. The quality and style of facilitation, including its sensitivity to context and support for local interpretation, is crucial for engaging potential participants [51, 52]. For example, feedback must be delivered constructively with an emphasis on continuous improvement and capacity rather than conveying connotations of surveillance or blame. [50, 53]

Mechanism 8. Persuasive leadership

As the 'holders' of an organisation's values, leaders are key agents in effecting workplace change in using evidence in health [54] and in policy [16]. Without strong visible leadership and a persuasively communicated 'vision' for change, interventions will be stymied [6]. Leaders' commitment to the intervention affects outcomes through multiple channels such as providing direction, shepherding the implementation and inspiring staff [15].

Leadership can be particularly important in encouraging receptivity to ideas that challenge practice-as-usual [3], especially when they have a 'transformational' (facilitative) rather than 'command and control' style of leadership [52]. However, the power of leadership support can be overstated: leaders' input is interpreted and is only one factor in many, so not even leaders' best efforts will necessarily secure enthusiasm [27].

Modeling refers to the way that people expand their knowledge and skills through observing others. This vicarious learning, particularly via leaders' behaviours, conveys organisational values and norms [20]. The status and prestige of external experts who model wider values and practices will enhance their impact. [30]

Mechanism 9. Strategic internal facilitation

Not all facilitators will implement the intervention as planned [2], or to the necessary extent [52]. Thus selection of people with attitudinal commitment to, and appropriate skills for this role, is a vital consideration [55, 56], yet this is an often overlooked part of implementation [2]. Facilitators may require substantial internal and external support as they wrestle with *"the compelling forces of fear of change, inertia, and investment in the status quo combine[d] with the inherently difficult and complex work of implementing something new"* [57].

In interventions, communication style, imagery and metaphor must be tailored for local participants [2], so effective marketing that taps into people's different motivations requires inside knowledge [45]. To function as change agents, internal facilitators must be credible advocates of the intervention values they are espousing, and receive support from managers [45, 56]. Researchers and policymakers often benefit from help in bridging their communication divide [58], particularly as disciplinary paradigms run deep, thus facilitators of research utilisation interventions may need to play an especially nuanced role in managing expectations, negotiation and problem-solving. [59]

References for Additional file 2.

1. Dobbins M, Cockerill R, & Barnsley J (2001) Factors affecting the utilization of systematic reviews. A study of public health decision makers. *International Journal of Technology Assessment in Health Care* 17(2):203-214.
2. Damschroder LJ, Aron DC, Keith RE, *et al.* (2009) Fostering implementation of health services research findings into practice: a consolidated framework for advancing implementation science. *Implementation Science* 4(1):1-15.
3. Greenhalgh T, Robert G, Macfarlane F, Bate P, & Kyriakidou O (2004) Diffusion of innovations in service organizations: systematic review and recommendations. *Milbank Quarterly* 82(4):581-629.
4. Conlan J, Grabowski S, & Smith K (2015) *Adult Learning*.Department of Educational Psychology and Instructional Technology, University of Georgia. http://epltt.coe.uga.edu/index.php?title=Adult_Learning. Accessed 7 Jul 2016.
5. Schein EH (2010) *Organizational culture and leadership* John Wiley & Sons.
6. Kotter JP (2012) *Leading change* Harvard Business Review Press, Boston, MA 2nd Ed.
7. Baron A (2012) What do engagement measures really mean? *Strategic HR Review* 12(1):21-25.
8. Tomm-Bonde L, Schreiber RS, Allan DE, *et al.* (2013) Fading vision: knowledge translation in the implementation of a public health policy intervention. *Implementation Science* 8(1):59.
9. Kothari A, Edwards N, Hamel N, & Judd M (2009) Is research working for you? Validating a tool to examine the capacity of health organizations to use research. *Implementation Science* 4(1):46.
10. Zimmerman B, Reason P, Rykert L, *et al.* (2013) Front-line ownership: generating a cure mindset for patient safety. *Healthcare Papers* 13(1):6.
11. Court J & Young J (2003) *Bridging research and policy: insights from 50 case studies. Working Paper 213*. Overseas Development Institute London.
12. Kluger AN & DeNisi A (1996) The effects of feedback interventions on performance: a historical review, a meta-analysis, and a preliminary feedback intervention theory. *Psychological bulletin* 119(2):254.
13. Srivastava UR & Singh M (2008) Psychological empowerment at the work place. *Global Journal of Business and Management* 2(1):53-73.
14. Rycroft-Malone J, Burton RC, Wilkinson J, *et al.* (2016) Collective action for implementation: a realist evaluation of organisational collaboration in healthcare. *Implementation Science* 11(1):1-17.
15. Fixsen DL, Naoom SF, Blase KA, & Friedman RM (2005) Implementation research: A synthesis of the literature.
16. Ng JL, Johnson A, Nguyen H, & Groth M (2014) *Workplace Culture Improvements: A Review of the Literature. A report for the Workforce Planning and Development Branch of the NSW Ministry of Health*. NSW Ministry of Health, Sydney.
17. Maxwell JA (2012) *A realist approach for qualitative research* Sage, Thousand Oaks, CA.
18. Trotter YD (2006) Adult learning theories: Impacting professional development programs. *Delta Kappa Gamma Bulletin* 72(2):8.
19. Aguinis H & Henle CA (2004) Ethics in research. *Handbook of research methods in industrial and organizational psychology*, ed Rogelberg S Blackwell, Oxford, pp 34-56.
20. Wood R & Bandura A (1989) Social Cognitive Theory of Organizational Management. *The Academy of Management Review* 14(3):361-384.
21. The Self-Determination Theory Group (2017) *Self-determination theory*. http://selfdeterminationtheory.org/. Accessed 5 Jan 2017.
22. IAP2 Australasia (2016) *The IAP2 public participation spectrum*.International Association for Public Participation (IAP2). https://www.iap2.org.au/resources/public-participation-spectrum. Accessed 12 Jul 2016.
23. Dobbins M, Hanna SE, Ciliska D, *et al.* (2009) A randomized controlled trial evaluating the impact of knowledge translation and exchange strategies. *Implementation Science* 4(1):61.
24. Nutley SM, Walter I, & Davies HT (2007) *Using evidence: How research can inform public services* The Policy Press.
25. Karanika-Murray M & Biron C (2013) The nature of change in organizational health interventions: some observations and propositions. *Salutogenic organizations and change*, eds Jenny GJ & Bauer GF Springer Netherlands.
26. Gabbay J & Le May A (2011) *Practice-based evidence for healthcare: Clinical mindlines* Routledge, Abingdon, Oxon.
27. Andersen H & Røvik KA (2015) Lost in translation: a case-study of the travel of lean thinking in a hospital. *BMC Health Services Research* 15:401.

28. Bandura A (1995) *Self-efficacy in changing societies* Cambridge University Press, New York
29. Merriam SB (2008) Adult learning theory for the twenty-first century. *New Directions for Adult and Continuing Education* 2008(119):93-98.
30. Manz CC & Sims HP, Jr. (1981) Vicarious Learning: The Influence of Modeling on Organizational Behavior. *Academy of Management Review* 6(1):105.
31. van Woerkom M, Nijhof WJ, & Nieuwenhuis LF (2002) Critical reflective working behaviour: a survey research. *Journal of European industrial training* 26(8):375-383.
32. Wholey JS, Hatry HP, & Newcomer KE (2010) *Handbook of Practical Program Evaluation* Jossey-Bass, 3rd Ed.
33. Greenhalgh T, Robert G, Bate P, Macfarlane F, & Kyriakidou O (2005) *Diffusion of innovations in health service organisations: a systematic literature review* Blackwell Publishing.
34. Weiner BJ (2009) A theory of organizational readiness for change. *Implementation Science* 4(1):67.
35. Lunenburg FC (2011) Self-efficacy in the workplace: Implications for motivation and performance. *International journal of management, business, and administration* 14(1):1-6.
36. McCray J, Turner H, Hall B, Price M, & Constable G (2014) Social care mentorship and employee engagement in the transformation of the Social Care Workforce. *Journal of Workplace Learning* 26(3/4):267-280.
37. Bullock A, Morris ZS, & Atwell C (2012) *A formative evaluation of the service delivery and organisation (SDO) management fellowships*. Final Report. NIHR Service Delivery and Organisation programme.
38. Edwards M & Evans M (2011) *Getting evidence into policy-making: parliamentary triangle seminar report*. in *ANZSIG Insights* ANZOG Insitute for Governance, University of Canberra.
39. Choi BCK, Pang T, Lin V*, et al.* (2005) Can scientists and policy makers work together? *Journal of Epidemiology and Community Health* 59(8):632-637.
40. Haynes A, Derrick GE, Chapman S*, et al.* (2011) From "our world" to the "real world": Exploring the views and behaviour of policy-influential Australian public health researchers. *Social Science & Medicine* 72(7):1047-1055.
41. Locally Driven Collaborative Projects program (2013) *Mapping A Pathway For Embedding A Strengths-Based Approach In Public Health Practice*. Public Health Ontario, Ontario.
42. Ivers N, Jamtvedt G, Flottorp S*, et al.* (2012) Audit and feedback: effects on professional practice and healthcare outcomes. *The Cochrane Library*.
43. Majone G (1989) *Evidence, argument, and persuasion in the policy process* Yale University Press, Yale University.
44. Dixon-Woods M, Leslie M, Tarrant C, & Bion J (2013) Explaining Matching Michigan: an ethnographic study of a patient safety program. *Implementation Science* 8:70.
45. McCormack B, Rycroft-Malone J, DeCorby K*, et al.* (2013) A realist review of interventions and strategies to promote evidence-informed healthcare: a focus on change agency. *Implementation Science* 8(1):107.
46. Jagosh J, Macaulay AC, Pluye P*, et al.* (2012) Uncovering the benefits of participatory research: implications of a realist review for health research and practice. *Milbank Quarterly* 90(2):311-346.
47. Dixon-Woods M, Bosk CL, Aveling EL, Goeschel CA, & Pronovost PJ (2011) Explaining Michigan: Developing an Ex Post Theory of a Quality Improvement Program. *Milbank Quarterly* 89(2):167-205.
48. Thompson GN, Estabrooks CA, & Degner LF (2006) Clarifying the concepts in knowledge transfer: a literature review. *Journal of Advanced Nursing* 53(6):691-701.
49. Best A, Greenhalgh T, Lewis S*, et al.* (2012) Large-System Transformation in Health Care: A Realist Review. *Milbank Quarterly* 90(3):421-456.
50. Sinuff T, Muscedere J, Rozmovitz L, Dale CM, & Scales DC (2015) A qualitative study of the variable effects of audit and feedback in the ICU. *BMJ Quality & Safety* 10.1136/bmjqs-2015-003978.
51. Gardner KL, Dowden M, Togni S, & Bailie R (2010) Understanding uptake of continuous quality improvement in Indigenous primary health care: lessons from a multi-site case study of the Audit and Best Practice for Chronic Disease project. *Implementation Science* 5(21).
52. Rycroft-Malone J (2004) The PARIHS Framework—A Framework for Guiding the Implementation of Evidence-based Practice. *Journal of nursing care quality* 19(4):297-304.
53. Hysong SJ, Best RG, & Pugh JA (2006) Audit and feedback and clinical practice guideline adherence: Making feedback actionable. *Implementation Science* 1(9):5-3.
54. McCormack B, Rycroft-Malone J, DeCorby K*, et al.* (2013) A realist review of interventions and strategies to promote evidence-informed healthcare: a focus on change agency. *Implementation Science* 8(1):107.
55. Damschroder LJ, Banaszak-Holl J, Kowalski CP*, et al.* (2009) The role of the champion in infection prevention: results from a multisite qualitative study. *Qual Saf Health Care* 18(6):434-440.

56. Haynes A, Butow P, Brennan S, *et al*. (2016) The pivotal position of 'liaison people': facilitating a research utilisation intervention in policy agencies. *Evidence & Policy* https://doi.org/10.1332/174426416X14817284217163.
57. Fixsen DL, Naoom SF, Blase KA, Friedman RM, & Wallace F (2005) *Implementation research: A synthesis of the literature*. National Implementation Research Network, University of South Florida, Tampa, Florida.
58. Newman J, Cherney A, & Head BW (2016) Do Policy Makers Use Academic Research? Reexamining the "Two Communities" Theory of Research Utilization. *Public Administration Review* 76(1):24-32.
59. Wehrens R, Bekker M, & Bal R (2010) The construction of evidence-based local health policy through partnerships: Research infrastructure, process, and context in the Rotterdam 'Healthy in the City' programme. *Journal of Public Health Policy* 31(4):447-460.
