# Supplementary material for: Policymakers’ experience of a capacity-building intervention designed to increase their use of research: a realist process evaluation
Source: Health Res Policy Syst. 2017 Nov 23;15:99. doi: 10.1186/s12961-017-0234-4 (PMC5701502; doi:10.1186/s12961-017-0234-4)
Supplement: Supplementary file 3 — Summary of SPIRIT intervention implementation fidelity. (PDF 1460 kb) [file 12961_2017_234_MOESM3_ESM.pdf]

Additional file 3 Summary of SPIRIT intervention implementation fidelity

Fidelity items	Agency 1		Agency 2		Agency 3		Agency 4		Agency 5		Agency 6	
CEO emails espousing the value of research	All sent by CEO		Some sent by liaison person		All sent by liaison person		Some sent by liaison person		All sent by CEO		All sent by CEO	
Use of systems for commissioning research	✓ Rapid review		✓ Evaluation plan		✓ Rapid review		✓ Linked data analysis		✓ Evaluation plan		✓ Rapid review	
Provision of tools and resources	✓		✓		✓		✓		✓		✓	
Essential elements[a] delivered in workshops:	Content	Interaction	Content	Interaction	Content	Interaction	Content	Interaction	Content	Interaction	Content	Interaction
- Introductory session	Extensive	Extensive	Extensive	Moderate	Extensive	Extensive	Extensive	Moderate	Extensive	Extensive	Extensive	Limited
- Audit & feedback 1	Extensive	Extensive	Extensive	Extensive	Extensive	Extensive	Extensive	Extensive	Extensive	Extensive	Extensive	Extensive
- Audit & feedback 2	Extensive	Extensive	Extensive	Moderate	Extensive	Moderate	Extensive	Extensive	Extensive	Extensive	Extensive	Moderate
- Skills seminar 1	Moderate	Moderate	Moderate	Moderate	Moderate	Extensive	Moderate	Extensive	Moderate	Moderate	Extensive	Extensive
- Skills seminar 2	Extensive	Extensive	Extensive	Extensive	Moderate	Limited	Extensive	Extensive	Moderate	Moderate	Extensive	Moderate
- Skills seminar 3	Extensive	Limited	Moderate	Limited	Moderate	Limited	Moderate	Moderate	Extensive	Extensive	Moderate	Extensive
- Leaders' program 1	Moderate	Limited	Moderate	Moderate	Moderate	Moderate	Moderate	Moderate	Moderate	Moderate	Unknown	Unknown
- Leaders' program 2	Extensive	Extensive	Extensive	Extensive	Extensive	Extensive	Extensive	Extensive	Extensive	Extensive	Extensive	Extensive
- Exchange forum 1	Extensive	Moderate	Moderate	Limited	Extensive	Limited	Extensive	Extensive	Extensive	Extensive	Moderate	Limited
- Exchange forum 2	Extensive	Moderate	Moderate	Extensive	Extensive	Extensive	Moderate	Moderate	N/A: e-bulletin chosen		Moderate	Extensive
- Exchange forum 3	Extensive	Extensive	N/A: e-bulletin chosen		N/A: e-bulletin chosen		N/A: e-bulletin chosen		N/A: e-bulletin chosen		N/A: e-bulletin chosen	
Total number of activities delivered	14		14		14		14		14		14	

[a]The essential elements for each workshop are clustered in two categories: *Content* includes the core topics, activities, messages and resources agreed with the agency, while *Interaction* includes how attendees participated in activities and, crucially, the extent to which they contributed to the content via discussion. Details about the methods and conduct of the fidelity assessment are provided elsewhere [59]
